# Multicolor strategies for investigating clonal expansion and tissue plasticity

**DOI:** 10.1007/s00018-021-04077-1

**Published:** 2022-02-20

**Authors:** L. Dumas, S. Clavreul, F. Michon, K. Loulier

**Affiliations:** Institute for Neurosciences of Montpellier (INM), Univ Montpellier, INSERM, Montpellier, France

**Keywords:** Transgenic mouse, Brain, Lineage tracing, Morphogenesis, Fluorescent reporters, Multichannel imaging

## Abstract

Understanding the generation of complexity in living organisms requires the use of lineage tracing tools at a multicellular scale. In this review, we describe the different multicolor strategies focusing on mouse models expressing several fluorescent reporter proteins, generated by classical (MADM, Brainbow and its multiple derivatives) or acute (StarTrack, CLoNe, MAGIC Markers, iOn, viral vectors) transgenesis. After detailing the multi-reporter genetic strategies that serve as a basis for the establishment of these multicolor mouse models, we briefly mention other animal and cellular models (zebrafish, chicken, drosophila, iPSC) that also rely on these constructs. Then, we highlight practical applications of multicolor mouse models to better understand organogenesis at single progenitor scale (clonal analyses) in the brain and briefly in several other tissues (intestine, skin, vascular, hematopoietic and immune systems). In addition, we detail the critical contribution of multicolor fate mapping strategies in apprehending the fine cellular choreography underlying tissue morphogenesis in several models with a particular focus on brain cytoarchitecture in health and diseases. Finally, we present the latest technological advances in multichannel and in-depth imaging, and automated analyses that enable to better exploit the large amount of data generated from multicolored tissues.

## Introduction

The advent of color has enriched the cinematic experience by highlighting the details of each scene, providing more information instantly to the viewer. In the same way, the discovery of the green fluorescent protein has revolutionized histology techniques by bringing color to scientific preparations. Most importantly, it has allowed deciphering of biological mechanisms within living tissue. However, the use of a single color for stem cell lineage tracking limits analysis to the scale of single cell or cell population, as it does not allow monitoring the dynamic properties of multiple neighboring cells concomitantly. To extend the scope of research to the multicellular scale, it is, therefore, necessary to label multiple adjacent stem cells with an extended palette of distinct markers. Labeling a tissue with several distinct fluorescent proteins (FP) helps to extract more information from a single tissue sample than single-color tracing approaches can do. In addition, multicolor strategies applied to trace the fate of multiple stem cells concomitantly enable the exploration of previously unapproachable developmental biology questions related to the heterogeneity of individual cell contribution to tissue homeostasis and morphogenesis. Here, we provide a comprehensive overview of current multicolor mouse models based on either classical (i.e. via pronuclear injection of linear DNA to generate transgenic animals, as in MADM, Brainbow and derivatives, or ifgMosaic) or acute (i.e. via in utero electroporation of plasmids in a targeted progenitor population, as in StarTrack, CLoNe, MAGIC Markers, iOn or viral infection using various vectors) transgenesis. Furthermore, we include a detailed description of their genetic design and a brief account of how their use provides key elements for understanding clonal expansion and tissue plasticity, essentially in the brain, but in other tissues as well. Finally, we discuss the latest imaging solutions, including two- and three-photon microscopy, tissue clearing, expansion microscopy and FACS applied to multicolor data sets, and briefly newly developed software tools for their automated analysis.

## Description of the multicolor mouse model strategies

**Fig. 1:**
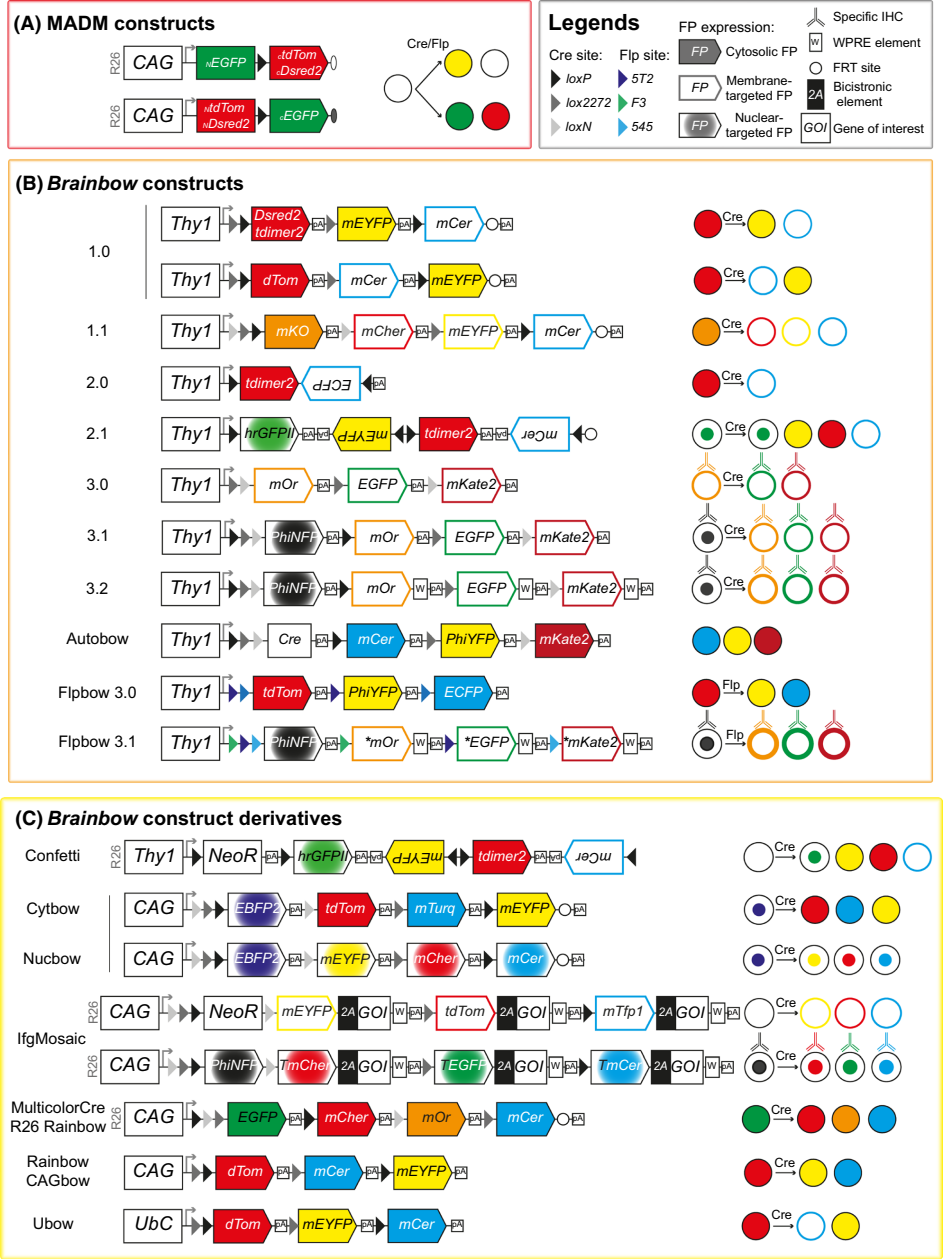
Scheme of different constructs used to generate multicolor mice: MADM (**A**), Brainbow (**B**) and Brainbow derivatives (**C**). The recombinase excises or inverts the DNA fragment between compatible target sequence with identical or opposite orientation, respectively. Compatible target sequence of Cre and Flp recombinase is pictured by pairs of triangles with the same color (loxP: black, lox2272: dark gray, loxN: light grey, 5T2: dark blue, 545: cyan, F3: green). The cell can express no FP (_C_EGFP, _C_tdTom, _C_Dsred2, _N_EGFP, _N_tdTom, _N_Dsred2, PhiNFP) or blue (EBFP2), cyan (ECFP, mCer, mTFP1, mTurq) yellow (mEYFP, EYFP, PhiYFP), green (_N_EGFP + _C_EGFP, hrGFPII, EGFP), orange (mKO, mOr) or red (mCher, _N_Dsred2 + _C_Dsred2, _N_tdTom + _C_tdTom, tdTom, tdimer2, mKate2) FP. To enhance the endogenous FP expression, WPRE element (W) has been added to Brainbow-3.2 and ifgMosaic constructs in addition to a Sumo start epitope (*) in Flpbow-3.1. The endogenous signal can be specifically amplified by immunostaining targeting antigenically distinct FP (Brainbow-3.0, 3.1, 3.2, Flpbow-3.1) or tags (T, ifgMosaic) as symbolized by an antibody scheme. Abbreviations used: *2A* bicistronic element, *GOI* gene of interest, *mCer* mCerulean, *mCher* mCherry, *mOr* mOrange2, *mTurq* mTurquoise2, *NeoR* neomycin resistance cassette, *pA* polyadenylation stop signal, *PhiNFP* non fluorescent mutant protein detectable after immunostaining, *dTom* dTomato, *tdTom* tdTomato, *T* tag (V5, His, HA), *W* WPRE element, * Sumo start epitope

**Fig. 2:**
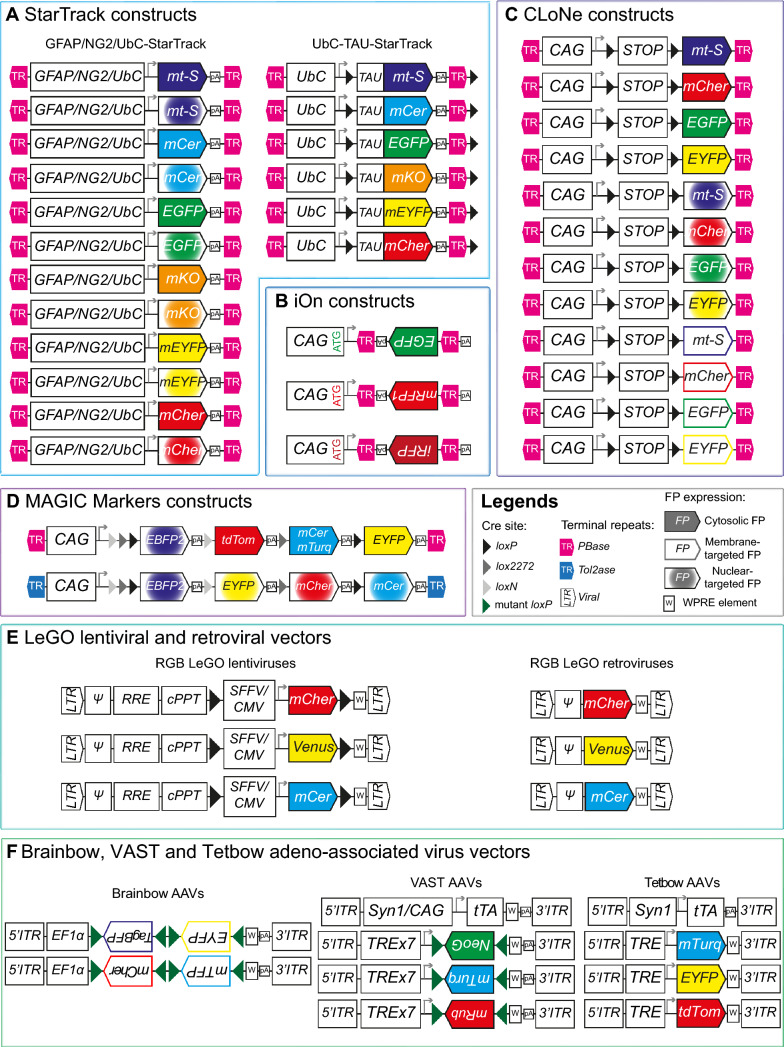
Scheme of constructs used to create acute transgenesis models such as StarTrack (**A**), iOn (**B**), CLoNe (**C**), MAGIC Markers (**D**), LeGO (**E**), Brainbow, VAST and Tetbow AAVs (**F**). Antiparallel terminal repeats frame the StarTrack, CLoNe, MAGIC Markers constructs while parallel terminal repeats are used in iOn plasmids. The endogenous signal is increased by inclusion of a WPRE (W) element in the LeGO, Brainbow, VAST and Tetbow viral vectors. Ubiquitous (CAG, CMV, SFFV, UbC), neuronal (Syn1) or glial (GFAP, NG2) promoters drive FP (co-)expression in the host cell and progeny after (co-)electroporation or (co-)transduction. Abbreviations used: *ATG* start codon, *CAG* composite promoter composed of the fusion of CMV enhancer, chicken ß actin promoter and rabbit ß globin splice acceptor site, *CMV* cytomegalovirus promoter, *cPPT* central polypurine tract, *GFAP* glial fibrillary acidic protein, *ITR* inverted terminal repeats, *TR* long terminal repeat used in viral constructs, *mCer* mCerulean, *mCher* mCherry, *mRub* mRuby2, *mt-S* mt-Sapphire, *mTurq* mTurquoise2, *NeoG* mNeonGreen, *NG2* neural glial antigen 2, *pA* polyadenylation stop signal, *PBase* piggyBac transposase, *RRE* rev response element, *SFFV* spleen focus-forming virus promoter, *Syn1* human Synapsin1, *tdTom* tdTomato, *TR* terminal repeats recognized by piggyBac (pink) or Tol2 (blue) transposases, *TRE* tetracycline response element, *tTA* tetracycline transactivator, *UbC* ubiquitin C promoter, *W* WPRE element, *Ψ* psi packaging signal

**Fig. 3:**
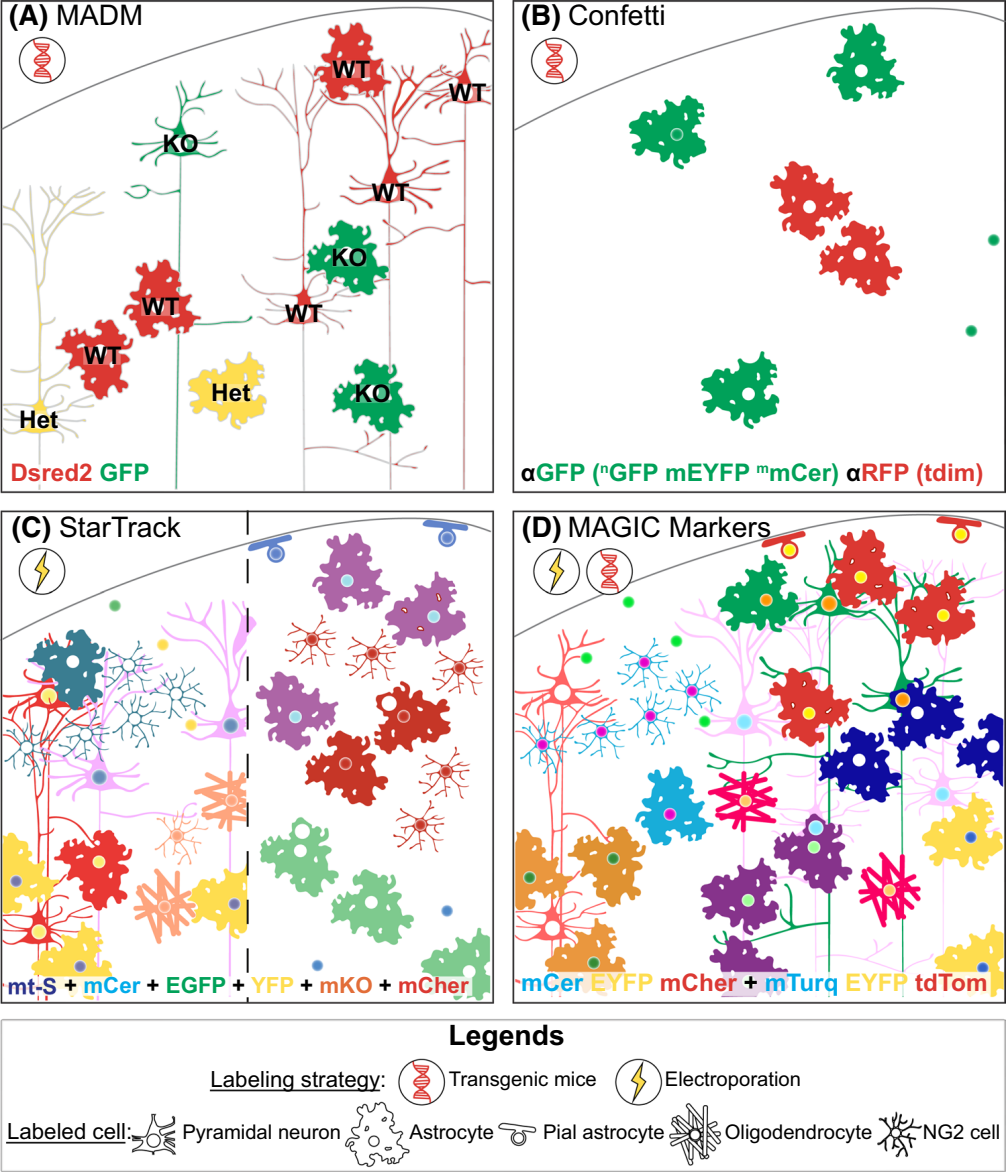
Typical labeling scheme of the main multicolor approaches used to study clonality in the mouse cerebral cortex. (**A**) Mosaic analysis with MADM labels mutant cells in green, heterozygous cells in yellow and wild type cells in red in order to both study clonal behavior and cell-autonomous gene function. (**B**) With a limited number of colors after signal amplification in the nervous system, the Confetti reporter mouse is suitable to follow slowly proliferating and poorly migrating cells, such as adult astrocytes. (**C**) StarTrack offers versatile tools to perform multiclonal analysis of brain development after electroporation of 12 integrative reporter vectors and transposase into neural progenitors driven by a ubiquitous UbC promoter (left side) or GFAP promoter (right side). (**D**) MAGIC Markers is useful for generating rare color codes that unambiguously identify clones such as sibling protoplasmic and fibrous astrocytes exhibiting a yellow nuclei and red cytoplasm after in utero co-electroporation with Cre recombinase and transposases. Abbreviations used: *KO* mutant green cell, *Het* heterozygous yellow cell, *WT* wild type red cell, *αFP (Fluorescent Protein)* signal after immunostaining against FP, *MADM* mosaic analysis with double markers, *MAGIC markers* Multiaddressable Genomic Integrating Color markers, *mCer* mCerulean, ^*m*^*mCer* membrane mCerulean, *mCher* mCherry, *mOr* mOrange2, *mt-S* mt-Sapphire, *mTurq* mTurquoise2, *NG2* neural glial antigen 2, ^*n*^*GFP* nuclear GFP, *tdim* tdimer2, *tdTom* tdTomato

The association of FP of distinct colors with the Cre/lox recombination system is the basis for most, if not all, multicolor mouse models. Whether it induces interchromosomal recombination (rare event, as in MADM, Table 1) or intrachromosomal recombination (more frequent event, as in Brainbow and derivatives, Table 2), the Cre/lox system is the cornerstone of most of multicolor models currently available. The stochastic events it triggers make it possible to multiply the color palette obtained from a small number of cassettes containing distinct FP coding sequences (Table 3). Two types of stochastic rearrangements can be induced during recombination. On one hand, MADM relies on the reconstitution of two FP coding sequences during mitosis and their subsequent expression in separate daughter cells. On the other hand, Brainbow and its derivatives depend on the random expression of one of 3–4 distinct FP whose coding sequences are inserted sequentially in the multicolor transgene (multiFP cassette in Brainbow scheme and its derivatives) in the recombined progenitor and its progeny (Fig. 1). When several copies of the multiFP transgene are present in the cell, the multiple choices of recombination will lead to the expression of a color resulting from the combination of several FP. The complexity of the color obtained is therefore related to the number of copies, which is itself dependent on the strategy of transgene integration (Fig. 2). Indeed, approaches based on MAGIC Markers in vivo electroporation [1] or classical transgenesis, such as the one used to generate the first generation of Brainbow mice [2], induce the insertion of several FP cassette copies and therefore generate an extended color palette (Fig. 3). On the contrary, the gene targeting approach (or knock-in) in the Rosa26 locus enables the integration of a single transgene copy, thus reducing the color palette to 3–4, corresponding to the FP included in the multiFP cassette (Confetti [3]). The choice of which multicolor approach to use depends on the biological question, the tissue model and the required number of distinct markers (Table 4).

**Table 1:** Inventory of MADM mice

References	Transgenic line names	Recombinase	Reporter construct	Recombined FP	Allele type	Integration locus	Addgene reference	JAX mice ID	Expression after crossing with
Zong et al. [4]	MADM-GR B6.129 or 129-Gt(ROSA)26Sortm3(CAG-EGFP/Dsred2)Luo/J	Cre	CAG	∅ or EGFP and/or DsRed2-Myc	Targeted, KI, FP	Gt(ROSA)26Sor, chromosome 6		006041 006075	MADM-RG and Cre lines
	MADM-RG B6.129 or 129-Gt(ROSA)26Sortm2(CAG-Dsred2/EGFP)Luo/J	Cre	CAG	∅ or EGFP and/or DsRed2-Myc	Targeted, KI, FP	Gt(ROSA)26Sor, chromosome 6		006067 006080	MADM-GR and Cre lines
Tasic et al. [6]	R26-GT Gt(ROSA)26Sortm6(ACTB-EGFP*,-tdTomato)Luo/J	Flp / Cre	ACTB	∅ or EGFP and/or tdTomato-Myc	Targeted, conditional, FP	Gt(ROSA)26Sor, chromosome 6	40025	017912	R26-TG and Cre/Flp lines
	R26-TG Gt(ROSA)26Sortm7(ACTB-EGFP*)Luo/J	Flp / Cre	ACTB	∅ or EGFP and/or tdTomato-Myc (R26-GT) or EGFP and non fluorescent tTA2 (R26G-tTA2)	Targeted, conditional, FP	Gt(ROSA)26Sor, chromosome 6	40026	017921	R26-GT or R26G-tTA2 and Cre/Flp lines
	R26G-tTA2 Gt(ROSA)26Sortm8(ACTB-EGFP*,-tTA2)Luo/J	Flp / Cre	ACTB	∅ or EGFP and non fluorescent tTA2	Targeted, conditional, transactivator,	Gt(ROSA)26Sor, chromosome 6	36878	017909	R26-TG and Cre/Flp lines
Hippenmeyer et al. [7]	MADM-7GT Iis5tm2.1(ACTB-EGFP,-tdTomato)Luo/J	Flp / Cre	ACTB	∅ or EGFP and/or tdTomato-Myc	Targeted, conditional, FP	Hipp7 / Igs5, chromosome 7		021457	MADM-7TG and Cre/Flp lines
	MADM-7TG Iis5tm1(ACTB-tdTomato,-EGFP)Luo/J	Flp / Cre	ACTB	∅ or EGFP and/or tdTomato-Myc	Targeted, conditional, FP	Hipp7 / Igs5, chromosome 7		021458	MADM-7TG and Cre/Flp lines
Tasic et al. [6]	Miya10GT Iis3tm2.1(ACTB-EGFP*,-tdTomato)Luo/J	Cre	ACTB	∅ or EGFP and/or tdTomato-Myc	Targeted, KO, FP	Miya10 / Igs3, chromosome 10		017923	Miya10TG and Cre lines
	Miya10TG Iis3tm1.1(ACTB-EGFP*)Luo/J	Cre	ACTB	∅ or EGFP and/or tdTomato-Myc	Targeted, KO, FP	Miya10 / Igs3, chromosome 10		017932	Miya10GT and Cre lines
Hippenmeyer et al. [5]	MADM-11TG Igs2tm2(ACTB-tdTomato,-EGFP)Luo/J	Flp / Cre	ACTB	∅ or EGFP and/or tdTomato-Myc	Targeted, KO, FP	Hipp11 / Igs2, chromosome 11		013751	MADM-11GT and Flp/Cre lines
	MADM-11GT Igs2tm1(ACTB-EGFP,-tdTomato)Luo/J	Flp / Cre	ACTB	∅ or EGFP and/or tdTomato-Myc	Targeted, KO, FP	Hipp11 / Igs2, chromosome 11		013749	MADM-11TG and Flp/Cre lines
Henner et al. [140]	MADM-ML-11GT Igs2tm1(ACTB-EGFP,-tdTomato)Zng/J	Flp / Cre	ACTB	∅ or EGFP and/or tdTomato-Myc	Targeted, KO, FP	Hipp11 / Igs2, chromosome 11		022976	MADM-ML-11TG and Cre/Flp lines
	MADM-ML-11TG Igs2tm2(ACTB-tdTomato,-EGFP)Zng/J	Flp / Cre	ACTB	∅ or EGFP and/or tdTomato-Myc	Targeted, KO, FP	Hipp11 / Igs2, chromosome 11		022977	MADM-ML-11GT and Cre/Flp lines
	MADM-ML-11GT/TG Igs2tm1(ACTB-EGFP,-tdTomato)Zng/ Igs2tm2(ACTB-tdTomato,-EGFP)Zng/J	Flp / Cre	ACTB	∅ or EGFP and/or tdTomato-Myc	Targeted, KO, FP	Hipp11 / Igs2, chromosome 11		030578	Cre/Flp line
Hippenmeyer et al. [7]	MADM-12GT Iis6tm2.1(ACTB-EFGP,-tdTomato)Luo/J	Flp / Cre	ACTB	∅ or EGFP and/or tdTomato-Myc	Targeted, KO, FP	John12 / Igs6, chromosome 12		021460	MADM-12TG and Cre/Flp lines
	MADM-12TG Iis6tm1.1(ACTB-tdTomato,-EFGP)Luo/J	Flp / Cre	ACTB	∅ or EGFP and/or tdTomato-Myc	Targeted, KO, FP	John12 / Igs6, chromosome 12		021461	MADM-12GT and Cre/Flp lines

*ACTB* chicken β actin promoter, *CAG* composite promoter composed of the fusion of CMV enhancer, chicken β actin promoter and rabbit β globin splice acceptor site, *Flp* flip recombinase, *ID* identiy number, *KI* knock-in, *KO* knock-out, *∅* no expression

**Table 2:**
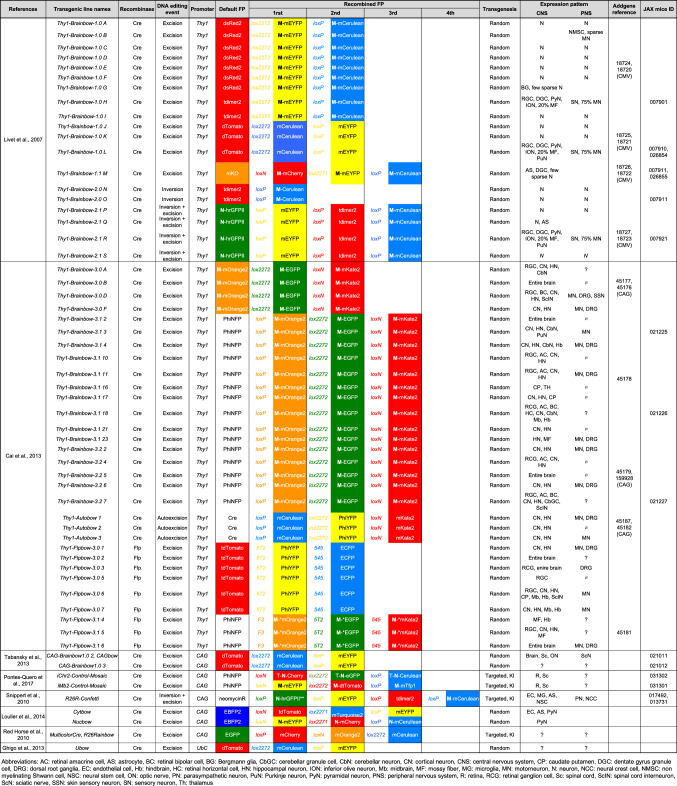
Inventory of Brainbow and derivative mice

*AC* retinal amacrine cell, *AS* astrocyte, *BC* retinal bipolar cell, *BG* Bergmann glia, *CbGC* cerebellar granule cell, *CbN* cerebellar neuron, *CN* cortical neuron, *CNS* central nervous system, *CP* caudate putamen, *DGC* dentate gyrus granule cell, *DRG* dorsal root ganglia, *EC* endothelial cell, *Hb* hindbrain, *HC* retinal horizontal cell, *HN* hippocampal neuron, *ION* inferior olive neuron, *Mb* midbrain, *MF* mossy fiber, *MG* microglia, *MN* motor neuron, *N* neuron, *NCC* neural crest cell, *NMSC* non myelinating Shwann cell, *NSC* neural stem cell, *ON* optic nerve, *PN* parasympathetic neuron, *PuN* Purkinje neuron, *PyN* pyramidal neuron, *PNS* peripheral nervous system, *R* retina, *RCG* retinal ganglion cell, *Sc* spinal cord, *ScIN* spinal cord interneuron, *ScN* sciatic nerve, *SSN* skin sensory neuron, *SN* sensory neuron, *Th* thalamus

**Table 3:**
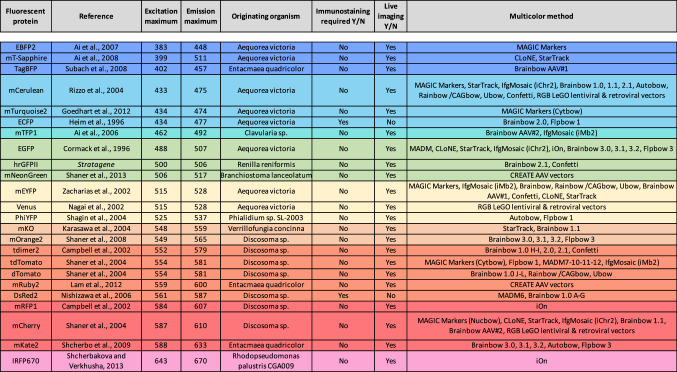
Fluorescent proteins used in multicolor mouse models: references and main features

**Table 4:**
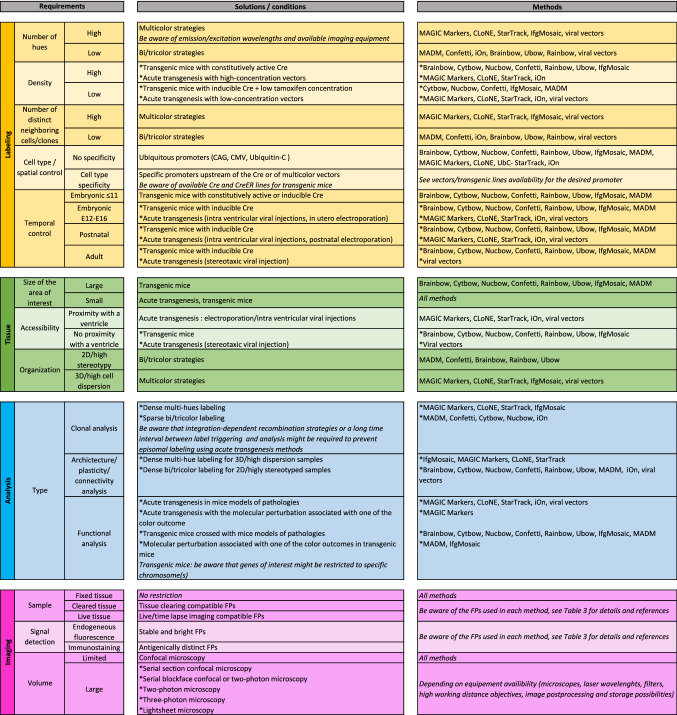
Decision support tool for choosing the best multicolor strategy in murine brain studies

### Transgenic mouse models: MADM, Brainbow and derivatives, ifgMosaic

#### MADM models

Mosaic analysis with double markers (MADM) was first described in 2005 [4]. This bi/tricolor strategy takes advantage of a Cre/lox system to induce clonal labeling and gene knock-out simultaneously in the mouse. Mutant homozygous labeled cells are generated in heterozygous unlabeled mice via interchromosomal mitosis recombination: two reciprocally chimeric genes containing part of the GFP and Dsred2 coding sequences, respectively (Fig. 1) are targeted by homologous recombination to the same loci of homologous chromosomes 6 in two different animals. When crossed, the resulting transheterozygous animals (heterozygous mice harboring reciprocal mutations on the same locus on homologous chromosomes) will produce a functional GFP and/or RFP upon Cre activity. Therefore, four outcomes are possible after mitosis: green or red, when two recombinant sister chromatids segregate into different daughter cells; yellow or unlabeled, when two recombinant sister chromatids segregate into the same daughter cells. The type, frequency, sparseness, and spatial pattern of cell labeling depend on the action of the chosen Cre that can be either constitutively active or inducible. Genetic modifications, initially restricted to genes located distal to the Rosa26 locus on chromosome 6 (later extended to chromosome 11 [5]) are obtained by inserting a mutation of interest distally to one of the chimeric gene knock-in sites. Three cellular genotypes are possible after mitosis: i) wild type (WT), ii) homozygous when two recombinant sister chromatids segregate into different daughter cells, red or green color reflecting the genotype, iii) heterozygous yellow cells when two recombinant sister chromatids segregate together. Subsequently, MADM was updated to improve signal detection. DsRed2, which initially required immunostaining, was replaced by tdTomato and modified to be used with more flexibility [6], and extended to chromosomes 10 [6], 7 and 12 [7] (Table 1). Very recently, Simon Hippenmeyer’s laboratory generated MADM transgenic mice for all remaining mouse autosomes by inserting MADM cassettes near their centromeres. This significantly expands the MADM toolbox to 19 chromosomes thus allowing to perform single-cell genetic mosaic analysis in more than 96% of the entire mouse genome [8]. MADM is suitable for clonal studies, qualitative and quantitative lineage tracing, and analysis of proliferation modes of progenitors at the single cell level (see “Multiclonal analysis findings in the mouse brain and other tissues” below). It allows genetic manipulation of candidate genes and their in vivo functional analysis during neural stem cell (NSC) lineage progression with the comparative analysis of neighboring control and mutant subclones [9]. In addition, MADM has been useful to evaluate recombination efficiency of specific Cre lines [10].

#### Brainbow models: Brainbow, Multicolor Cre/ Rosa26 Rainbow, Rainbow/ CAGbow, Cytbow and Nucbow, Ubow, Confetti, Autobow

As the breakthrough drawings of Cajal a century ago, Brainbow revolutionized our visualization of the nervous system through color [2]. Because of the multiple colors (reminiscent of a rainbow) expressed in the nervous system (aka “brain-bow”) and used to study its connectivity, "Brainbow" has opened up a whole new area of tissue visualization at a multicellular scale [2]. Since then, new versions of this methodology have been developed (Confetti, Rainbow, Ubow…; Fig. 1) to decipher key biological phenomena regarding stem cell lineage tracing and dynamic behaviors, homeostasis of tissues undergoing renewal or repair, and cellular mechanisms involved in organogenesis at the level of multiple individual cells in animals or plants [11]. The Brainbow strategy is based on stochastic recombination of a transgene comprising 3 to 4 spectrally distinct FP flanked by 2–3 pairs of palindromic nucleotide sequences called "lox sites". The recombination of the multiFP transgene is triggered by random recognition of one of the pairs of lox sites by the Cre recombinase enzyme. Depending on the orientation of the paired lox site sequences (identical as in Brainbow-1.0, 1.1, 3.0, 3.1, 3.2, opposite as in Brainbow-2.0, or both as in Brainbow-2.1), the recombination will lead to either the excision (Brainbow-1.0, indelible labeling) or the inversion (Brainbow-2.0, reversible labeling strategy) of the FP DNA sequence located between the two identical lox sites. Prior to recombination, only the first FP coding sequence downstream of the neuronal promoter Thy1 is expressed by default (DsRed, tdTomato or tdimer2 in Brainbow-1.0 and 2.0; monomeric Kusabira Orange (mKO): Brainbow-1.1; nuclear GFP: Brainbow-2.1; mOrange2: Brainbow-3.0). Brainbow mice are generated by pronuclear injection resulting in tandem integration of multiple copies of the multiFP transgene into the genome. As the Cre recombinase is editing each copy of the Brainbow transgene independently of one another, the genomic integration of multiple copies leads to the combinatorial expression of distinct FP.

Several improvements of the initial Brainbow lines have been made to facilitate imaging and consecutive analysis [12] (Table 2). In addition to Rosa26 reporter lines, some Brainbow transgenes (Brainbow-1.0, 1.1, 2.1) also contain a FRT site in order to reduce tandem transgene copy number and thus color diversity after Flp-mediated recombination [2]. This exclusive expression of FP is helpful for studying tissues with low cell mixing or renewal and eases considerably the image analysis. To keep the ability of fast tissue screening while offering a larger color palette to recombined cells, an alternative Brainbow version was developed in which the default red FP of the initial Brainbow mice was replaced by a stop cassette and a nuclear non fluorescent mutant protein (called PhiNFPnls) only detectable after immunostaining (Brainbow-3.1, 3.2; [12]). The initial FP have also been upgraded from spectrally different but antigenically similar (mEYFP, mCerulean and hrGFPII in Brainbow-1.0, 1.1 and 2.1) to both spectrally and antigenically distinct (mOrange2, EGFP and mKate2 in Brainbow-3.0, 3.1, 3.2 and Flpbow-3.1) offering the possibility to specifically detect each FP using immunostaining [12].

**Fig. 4:**
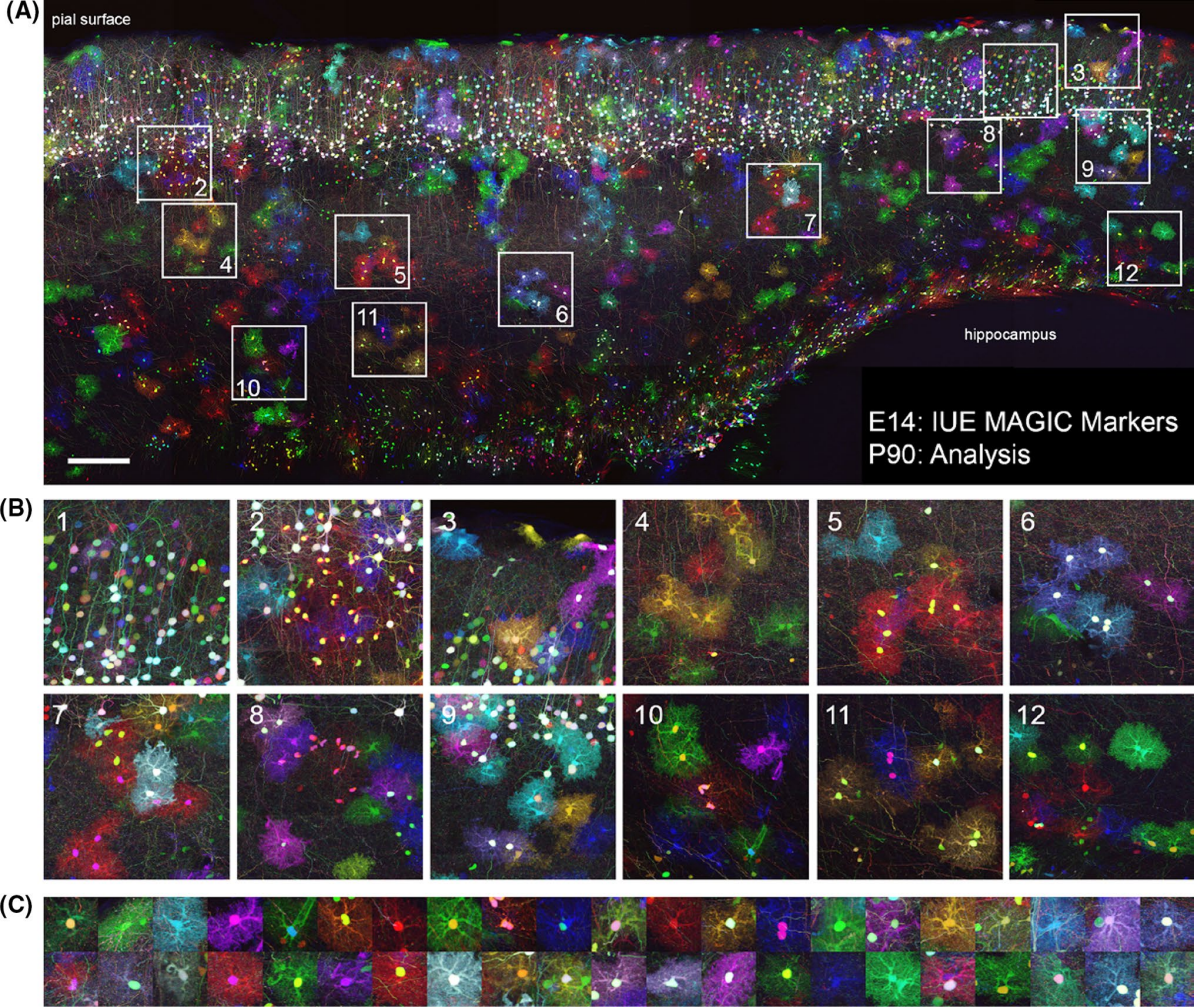
MAGIC Markers labeling in the adult mouse cortex enables multicolor clonal analysis. (**A**) Maximal projection of 80 μm thick sagittal section displaying the cerebral cortex of a 3 month-old mouse. Cortical progenitors were targeted at E14 by in utero co-electroporation of MM Nucbow and Cytbow, their respective transposases Tol2 and piggyBac, and a self-excisable Cre to trigger multicolor labeling. Neurons born at the time of electroporation express a wide palette of colors resulting from the expression of FP from both integrated and episomal vectors and have migrated to the upper cortical layers, while the oligodendrocyte and astrocyte progenies, which have colonized the entire cortex, have diluted episomes and only express integrated, stable, and clonally reliable markers. Mosaic image stacks were obtained by sequential confocal acquisition of mTurquoise2/mCerulean, mEFYP, mCherry/tdTomato (rendered in blue, green and red, respectively), with a 20 × 0.8NA oil objective on an Olympus FV1000 microscope, and stiched with Fiji. (**B**) Close-ups of neurons (first panel), oligodendrocytes and astrocytes clusters. (**C**) Examples of cytoplasm and nucleus-cytoplasm color combinations of cortical glial cells found in this one 80 μm section. *IUE* in utero electroporation. Scale bar: 200 μm

Brainbow was initially designed to map the neuronal network architecture [2], but after successive refinements, the application range of Brainbow and its derivatives has expanded beyond connectomics alone. In addition to mouse lines expressing colors in mature neurons [2, 12], transgenic mice generated from Brainbow transgenes and displaying a broader expression have emerged, which include Multicolor Cre reporter or Rosa26 Rainbow [13, 14], Autobow [12], Rainbow or CAGbow [15, 16], Ubow [17], Cytbow and Nucbow [1] mouse lines, and the most used outside of the central nervous system: the Confetti mouse [3]. The replacement of the original neuronal Thy1 promoter by the broadly active CAG or the strong Ubiquitin C promoters was a stepping-stone to study clonal relationships using multicolor approaches. The Multicolor Cre reporter or Rosa26 Rainbow mouse is a simplified cytoplasmic version of the Brainbow-1.1 mouse in which the neuronal promoter is changed for a CAG promoter and the sequence of FP is switched from mKO, membrane-targeted mCherry, membrane-targeted EYFP and membrane-targeted mCerulean to EGFP, mCherry, mOrange and mCerulean [13, 14]. This knock-in mouse harboring a maximum of 10 colors [18] has been used so far to examine the clonal contribution to coronary development [13], digit tip regeneration [14] or intestinal cancer [19]. The Rainbow transgenic mouse expresses the Brainbow-1.0 construct under the control of the CAG promoter and was originally used to show that inequality between blastomeres is initiated as early as the 4-cell stage [15]. Generated through classical transgenesis and therefore independent of the Rosa26 locus, Rainbow (CAGbow) mouse line expresses up to 21 color cues in homozygous animals and the Brainbow-1.0 transgene is detected in many tissues including the nervous system [15, 20]. Jean Livet’s laboratory further expanded the Brainbow-1.0 combinatorial palette by addressing the expression of the distinct FP to different subcellular locations (cytoplasm, nucleus, membrane, mitochondria) to unambiguously track multiple clones in densely labeled tissues. These Multiaddressable Genomic Integrating Color (MAGIC) Markers can be used directly following in vivo delivery using acute transgenesis (see below and Fig. 4) and have been used to generate new transgenic lines called Cytbow and Nucbow which ubiquitously drive the Cre-dependent multicolor labeling in the cytoplasm and nucleus, respectively [1]. Designed on the Brainbow-1.1 template with three excision events, these CAG-driven transgenes express a nuclear EBP2 by default and Cre recombinase triggers the compartmented expression of three FP (Cytbow: tdTomato, mTurquoise2, mEYFP and Nucbow: mEYFP, mCherry, mCerulean). Brainbow transgenic approaches provide a maximum of color diversity from a single transgene and have been declined in many different versions. “Ubow” transgenic mice were generated using the Brainbow-1.0L cassette including 3 distinct FP (dTomato, mEYFP and mCerulean) flanked by pairs of incompatible lox sites placed in the same orientation. The main difference with the first generation of Brainbow mice is a replacement of the Thy1 neuronal specific promoter by the strong human Ubiquitin-C (UbC) promoter that enables a broad expression of the FP in various tissues, including the mouse immune system [17]. Ubow model is compatible with the use of both constitutive and inducible Cre lines and therefore, a powerful tool to perform multicolor fate mapping.

To elucidate the self-renewal dynamics of multiple stem cells, Snippert and colleagues generated the Confetti mouse by inserting the Brainbow-2.1 transgene preceded by a floxed unit combining a neomycin resistance gene (Neo^R^) and a stop signal under control of the exogenous CAG promoter in the Rosa26 locus [3]. The Brainbow-2.1 construct is composed of two invertible floxed modules organized in tandem offering three inversion and two excision recombination events (Fig. 1). Closest to the promoter, the neomycin resistance cassette is expressed by default and the downstream polyadenylation signal is used as transcriptional roadblock to prevent FP expression without active Cre recombinase. The activated Cre recombinase catalyzes the excision of this stop cassette resulting in the random expression of either a nuclear GFP, mEYFP, tdimer2 or a membrane-targeted mCerulean. However, due to the targeted transgenesis method used to generate this multicolor knock-in, only a single copy of the transgene was inserted in the Rosa26 locus. Therefore, the palette of color expressed after recombination is limited to one of the 3-4 possible FP in hemizygous animals, reaching up to 10 distinct color combinations in homozygous mice [21–23]. However, multiple reports indicate that nuclear GFP + cells are underrepresented in several organs of the Confetti mouse [3, 21, 23–25]. In the initial publication, long-term fate mapping of individual stem cells in Confetti; Ah-Cre mice after *β*-naphthoflavone exposure shows that intestinal crypt drifts almost completely from multiclonal towards monoclonal composition in 30 weeks [3]. Interestingly, the recombination in Ah-Cre line used in this study is tamoxifen-independent and is based on the upregulation of the CYP1A1 promoter activity through the nuclear translocation of the endogenous AhR after xenobiotic exposure. Since its generation, Confetti reporter mice have been broadly used to trace lineage in multiple conditions, such as intestinal adenomas [26], skin wound healing [27], breast cancer [28] and also in the aging [24, 29] and injured nervous system [30, 31]. Furthermore, this model has been used to study lacrimal gland epithelium expansion [32]. In this study, a constitutive Cre was used to label as many cells as possible. Because of the continuous recombinase activity, cells were sorted into two populations, depending on the first recombination event: GFP and/or YFP expressing cells, and RFP and/or CFP expressing cells. This approach helped to visualize cell intercalations between the two cell populations.

#### IfgMosaic

Pontes-Quero and colleagues developed an elegant genetic strategy for the conditional induction of mosaic gene expression based on the expression of distinct and compatible FP: inducible, fluorescent, and genetic mosaic (ifgMosaic) [33], reviewed in [34]. This system, which can lead up to 15 distinct clone colors, relies on the combination of Cre/lox recombination and knock-in insertions in the mouse Rosa26 locus of multiFP transgenes, is compatible with live imaging and immunostaining, and has the possibility to insert a mosaic co-expression of other genes of interest as well (Fig. 1). Two constructs enable a combinatorial labeling in two subcellular compartments: iMb-Mosaic (three membrane-localized FP) and iChr-Mosaic (three chromatin-localized FP). IfgMosaic was validated in vitro in ES cells and in vivo in mice before applying it to gene function analysis to study angiogenesis and neurogenesis [33]. However, ifgMosaic plasmids are difficult to clone, amplify and target to the Rosa26 locus due to their size and repetitive sequences. To overcome this issue and rapidly obtain single copy experimental ifgMosaic animals, Pontes-Quero and colleagues designed two new systems. Despite a limited output for in vitro expression, the PB-Rosa26 BAC system was efficient for the generation of transgenic animals. The second system relies on CRISPR/Cas strategy to enhance homologous recombination and the efficiency of gene targeting, which was validated in vitro and used to generate Rosa26 ifgMosaic mouse lines with genetically improved versions of ifgMosaic constructs (iMb2-Mosaic and iChr2-Mosaic) (Table 2). The latter were also modified with FRT sites to be recombined with Flpo or Flpo^ERT2^ to allow two distinct recombination events sequentially, allowing cell-autonomous and non-cell-autonomous gene function analysis up to 6 genes simultaneously [33].

### Acute transgenesis models: StarTrack / CLoNe / MAGIC Markers / iOn / viral vectors

#### StarTrack and derivatives

This method is based on the in utero electroporation of 12 integrative plasmids, each expressing one FP reporter (mT-Sapphire, mCerulean, mEYFP, EGFP, mKO, and mCherry) driven by the astrocytic GFAP (StarTrack [35–37]), oligodendroglial NG2 (NG2-StarTrack [38, 39]) or the ubiquitous Ubiquitinase C (UbC-StarTrack [40, 41]) promoter which results in the combinatorial expression of distinct FP in two subcellular compartments (nucleus and cytoplasm) of the targeted cells (Fig. 2). The co-electroporation of this mixture containing integrative reporter transgenes along with a piggyBac transposase-expressing plasmid [42] enables the integration of multiple FP coding sequences that generate a combination of FP markers, which will be shared by sister cells. Therefore, StarTrack constitutes a useful and versatile tool to perform multiclonal analysis and lineage tracing during neural development.

#### CLoNe

Also based on multiple integrating vectors that carry each a single FP coding sequence (mT-Sapphire, EGFP, mEYFP or mCherry) behind a floxed stop sequence, the CLoNe (clonal labeling of neural progenies) strategy takes advantage of the Cre/lox system to trigger the expression of each of these multiple labeling vectors in distinct subcellular compartments (nucleus, cytoplasm, membrane) of neural progenitors through the action of Cre recombinase controlled by various promoters (CAG, Emx2, Dlx1/2, Fig. 2; [43]). Using this technique, the authors obtained a multicolor labeling of progenitors in the mouse cerebral cortex and chick pallium after co-electroporation of vectors expressing the coding sequences of CLoNe transgenes, piggyBac transposase (PB) and Cre recombinase. Following the expression of the Cre recombinase driven by the Emx2 promoter, the resulting astrocyte clonal labeling revealed that astrocytes form clusters of cells with dispersion variability. In a second study, the authors used this strategy in combination with a Tbr2Cre mouse line to show that intermediate progenitor cells contribute to the glutamatergic neurons of all cortical layers where the clonally related cells cluster closer than unrelated cells [44].

#### MAGIC markers

MAGIC markers (MM) strategy was designed after Brainbow-1.1 to obtain a high number and equilibrated distribution of stable labels, whose expression timing and sparseness can be controlled. It relies on a minimum number of integrative vectors (available with PB and Tol2 transposition systems) and 4 spectrally distinct FP whose coding sequences are flanked by three pairs of incompatible lox sites (Fig. 2; [1]). Four constructs expressing cytoplasmic (Cytbow), nuclear (Nucbow), membrane (Palmbow), or mitochondria (Mitbow)-targeted labels under the control of a CAG promoter can be recombined through the controlled action of Cre recombinase using either a floxed Cre construct (self-excisable) or specific Cre mouse lines, inducible or not. Following the in utero electroporation of MM, Cre recombinase and PB/Tol2 transposases, the nuclear EBFP2 is expressed by default and Cre-recombined cells express either tdTomato/ mCherry, mEYFP, and mCerulean/ mTurquoise2 alone (from a single copy) or in combination (from multiple copies), leaving the far-red channel available for additional immunolabeling to further characterize the MM-labeled cells. This approach, which does not require immunostaining for the detection of MM FP expression, was validated in the mouse cortical progenitors as well as in the chick spinal cord and retina. It allows multicolor visualization of fixed or live tissues with standard confocal or multiphoton microscopies [1, 45–47] and is applicable to disrupt specific signaling pathways in a mosaic manner, following the association of one of the color outcomes to a specific molecular perturbation [1].

#### iOn

Stable genomic integration of a reporter into electroporated neural progenitor cells is an essential prerequisite to trace their entire progeny, from neurons to glial cells. However, transient episomal expression leads to an artifactual burst of reporter expression that was, until recently, undistinguishable from expression provided by the integrated transgenes. To overcome this hindrance, Kumamoto and colleagues developed an integration-coupled On genetic switch (iOn) strategy that triggers FP expression solely upon integration of trichomatic iOn vectors encoding GFP, RFP and iRFP into the host genome (Fig. 2; [48]). Each iOn vector has parallel terminal repeats recognized by PB transposase allowing the promoter and gene of interest to be reunited and expressed only upon transposition. Multiplexed stable transgenesis of HEK and human iPS cells with iOn vectors has been established in vitro without drug selection. In addition, iOn strategy was used to analyse in vivo the lineage and functional mosaicism of spinal cord and retinal progenitors in electroporated chick embryos. The iOn multicolor tag strategy constitutes therefore a direct somatic transgenesis readout that is faithfully passed to the whole progeny upon cell division [48].

#### Viral vectors

Besides electroporation, acute somatic transgenesis can be accomplished through viral gene delivery. Due to the limited packaging capacity of most viral vectors (transgene size limit: lentivirus and γ-retrovirus up to ~ 9 kb, adeno-associated virus (AAV) ~ 4.7 kb), the classical trichromatic construct has been split into several transgenes each encoding from one [49–52] to two distinct FP [12]. So far, four different types of viral vectors have been used to recreate a multicolor labeling: lentiviral [49–51], γ-retroviral [52], AAV [12], pseudorabies viral [53] vectors.

##### LeGO lentiviral and retroviral vectors.

Both lentivirus and γ-retrovirus are reliable for long-term clonal analysis via the permanent integration of the transgene into the host’s genome (Fig. 2). Weber and colleagues created a novel set of eleven lentiviral gene ontology (LeGO) vectors driving notably the expression of a wide panel of FP [49, 50]. Each of these third-generation vectors comprised a broad SFFV promoter and several options leading to the expression of either a FP alone or along with a gene of interest and/or a short-hairpin RNA within one single cell. Facilitating gene function analysis through the misexpression of potentially interacting genes, LeGO vectors have been reported to force co-expression of two genes (e.g. Olig2 and Nkx2.2) after sequential transduction of primary neural stem cells in vitro [49]. Several adaptations of the standard LeGO vector exist including a series of new drug-selectable FP vectors to select labeled cells independently of FACS [50] and barcoded LeGO-vector libraries [54]. Furthermore, the insertion of ubiquitous CAG [50] or CMV [52] promoters into LeGO vectors equipped with drug-selectable FP expands the field of potential applications, including clonal analysis. Indeed, LeGO vectors ensure stable and efficient expression of the carried transgene in antibiotic-selected neural stem cells and their neuronal and astrocytic progeny in vitro [50]. Of note, the new SFFV/CAG-FP-gene of interest or resistance-shRNA cassette present in LeGO vector deriving from the Lentilox 3.7 is floxed and therefore removable in any Cre^(ERT/T2)+^ environment [49, 50]. RGB marking was then created through the simultaneous transduction of three LeGO vectors encoding mCherry, Venus and Cerulean, creating 63 theoretically different cues and applied to track multicolor clones in vitro and in vivo during liver regeneration and tumorigenesis [51]. Following the same primary color mixing approach to generate multicolor hues, three γ-retroviral vectors encoding mCherry, Venus and Cerulean were created using the SFFV promoter and ecotropic envelope and were employed to trace hippocampal neurogenesis [52].

##### Adeno-associated viral vectors (Brainbow, VAST and Tetbow).

The Brainbow approach has been adapted to AAV vectors [12] and two versions of Brainbow AAV serotype 9 vector currently exist. Each construct contains two farnesylated FP (TagBFP and mEYFP or mCherry and mTFP1) placed between variant lox sites. Without Cre recombinase, no FP is expressed as the dual FP cassette is placed in reverse orientation. Upon Cre action, either the first FP is expressed or the second one or none. Recombined pairs of Brainbow AAVs can result in at least 8 different color outcomes [12] (Fig. 2). Studies using Brainbow AAVs have, for example, demonstrated the convergence of the retinogeniculate pathway [55] and subclassified molecularly defined interneurons on their morphologies [56]. Alternatively, multicolor labeling is achievable through two AAV-based Tet-Off systems called VAST [55] and Tetbow [57] (Fig. 2). Each system relies on the co-transduction of four AAVs, one vector expressing a tetracycline transactivator (tTA) inducer and three different inducible FP encoding vectors (VAST: mTurquoise2, mNeonGreen, tdTomato/ Tetbow: mTurquoise2, EYFP, tdTomato). Both approaches improve color variety independently of the labeled cell density. This is achieved by expressing a moderate level of tTA, either via a viral intersectional system in which reporter expression depends on both the inducer co-transduction and Cre action (VAST, [55]) or simply by diluting the inducer vector (Tetbow, [57]). In addition to conventional injection, it is now possible to efficiently label the central or peripheral nervous system directly through the vasculature using the VAST system’s vectors exhibiting customized capsids [54, 55]. The color chart created by AAV-based Tet-Off approaches allows tracing of individual axons of multiple mitral/tufted cells in the olfactory system [57] and cholinergic neurons in the intestine over several millimeters [55]. Although AAVs infect both dividing and non-dividing cells, they are, however, non-integrating vectors that will be progressively diluted and ultimately lost with successive divisions. As AAV-based reporters do not retrace the entire fate of dividing mother cell progenies, which therefore precludes their usage in clonal analyses, their application is mostly restricted to study tissue architecture or cellular morphology.

##### Rainbow pseudorabies viruses.

The functional organization of neural circuits can be uncovered with retrograde pseudorabies virus. Boldogkoi and colleagues engineered a set of Rainbow pseudorabies virus strains expressing distinct FP (mCerulean, ECFP, mTFP1, EGFP, EYFP, mKo1, Dsred2, mCherry) to reveal the organization of connected neurons among spatially intermingled ones [53]. Two pseudorabies viruses have been designed to create a conditional multicolor label to identify only collateralized neurons [58]. Both in the US4 locus, PRV-263 virus carries the Brainbow-1.0L cassette under the CMV promoter while PRV-267 contains the Cre recombinase associated with a nuclear VP26-mRFP highlighting only the infected neuron. Dual infection of both kidneys with either PRV-263 or PRV-267 labels collateralized neurons and the polysynaptic circuit innervating both kidneys [58].

## Main other multicolor models

Besides their utility in mice, multicolor approaches have been mainly employed in zebrafish, chicken, drosophila and human iPSC models.

### Zebrafish

Zebrabow was the first multicolor zebrafish described [59]. It can be either used acutely via injections of Brainbow DNA [59, 60] or integrated to the genome in a stable transgenic line [60, 61]. In the original Zebrabow, the CMV and Ubiquitin promoters, for ubiquitous labeling, or Islet1, for trigeminal sensory neuron specificity, drive three FP flanked by two pairs of incompatible lox sites. dTomato FP is expressed by default and Cre expression mediated by a transgenic Cre fish triggers two possible recombinations resulting in the expression of Cerulean or YFP [59]. Zebrabow expression was later rendered GAL4-inducible (UAS::Zebrabow) to allow broad or tissue specific labeling and axon process tracing. It was validated in the cornea, the nervous system and other organ systems [61] and made compatible with gene manipulation using far-red reporters [62]. Moreover, Zebrabow is a valuable tool to visualize the developing nervous system in vivo using time-lapse confocal microscopy and thereby study cell proliferation and apoptosis at the clonal level in the brain [63, 64]. Zebrabow was otherwise used for clonal analysis in the embryonic Meckel’s cartilage [65], palate [66], retina [67], gut [68], and pancreas [69]. Other multicolor fish models have been developed to study embryogenesis in various organs. PriZm is a multicolor fish model based on Brainbow-1.0L that can be used to probe heart morphogenesis [70, 71]. Skinbow, based on Brainbow-1.0, is designed to study long-term surveillance of epithelial regeneration [72]. Finally, adapted from Brainbow-1.1 to prevent any labeling by default, Multibow has its color expression triggered by heat-shock inducible and tissue specific Cre driver lines. It relies on multiple transgenes to express 7 different FP in three subcellular compartments (cytoplasm, membrane and nucleus) and a Tol2 transposition system for transgene integration in an acute context [73]. The method was applied to perform time-lapse imaging of craniofacial development and to study tissue regeneration.

### Chick

The chick has been utilized as proof of concept for several genetic multicolor tools and imaging systems because of the easy access to the spinal cord and retina by in ovo electroporation, and the known developmental stereotypy of these structures. First, the mosaic expression obtained with in ovo electroporation of pCAGGS-Brainbow-1.1M was used to discriminate axonal projections during calyx-type synaptic development in the ciliary ganglion [74]. In another study, MAGIC Markers were validated in the developing chick retina and spinal cord by in ovo electroporation and used to reveal the clonal patterns of neighboring neural progenitors with standard microscopy and time-lapse imaging [1]. In addition, the chick model was employed to validate the CLoNe cocktail of vectors used to observe relationships between neighboring clones [43]. Later on, iOn has been also validated in the chick. It enables reliable lineage tracing in the spinal cord and retina, and functional color-coded mosaic perturbations to investigate non-cell-autonomous effects on unperturbed clones [48]. Moreover, fixed chick embryo Brainbow samples were employed to validate a novel simultaneous four-channel optical epifluorescence microscopy based on simple wedge prisms [75]. Using this method, the authors identified individual neurons by their spectra without extensive image postprocessing.

### Drosophila

Opening the way to MADM strategies, the mosaic analysis with a repressible cell marker (MARCM) system [76] was developed in drosophila to generate mosaic organisms with mutant cells labeled in green (mCD8-GFP) and WT cells in red (MYC). MARCM enabled coexpression of both transgene of interest and marker gene in mosaic clones, allowing functional analysis and developmental lineage tracing by controlling the low occurrence of a Flp/FRT-mediated interchromosomal recombination. miRNA-based twin-spot MARCM was later designed to improve MARCM by labeling both daughter cells generated after interchromosomal recombination with distinct colors, increasing the resolution of lineage analysis, as shown in the Drosophila olfactory learning and memory center [77].

After the Brainbow strategy was developed in mice, several models of multicolor flies have been generated. Three models, dBrainbow [78], Flybow [79] and LOLLIbow [80], are based on the Brainbow-1, Brainbow-2 and Brainbow-1.1 systems, respectively. dBrainbow enables the distinction of 6 separable colors (2 copies, with mTFP1.0, EGFP and mKO2). The resulting UAS-dBrainbow flies were used to visualize multiple neuron lineages and trajectories of individual neuron projections after Cre recombination. This enabled the mapping of neuron circuitry using live imaging of endogenous fluorescence for EGFP and mKO2, or immunostaining of fixed tissues for the entire color hue. Flybow relies on a modified version of Brainbow-2 based on a Flp-FRT recombination system more compatible with functional analysis in the fly, and selected membrane EGFP, mCerulean and mCitrine and mCherry for the FB1.1 construct. Flybow was tested in the visual system to visualize neurons, glia and epithelial cells with 4 distinct colors. An upstream Flp-out cassette (Lamin-2xHA) was added in FB2.0 construct to enable FP expression via excision of the stop cassette, therefore resulting in a sparse cell labeling [79]. Flybow was later applied to trace individual embryonic glial cells during larval development and analyze their morphology [81] and to study the morphological characteristics of single clock neurons of the fly circadian system at the anatomical level [82]. LOLLIbow is a live imaging system allowing the observation of fine morphological details of individual cells where the timing of the membrane-targeted stochastic tricolor cell labeling is controlled with a blue light pulse thanks to a light-inducible DNA-editing Cre recombinase [80].

Another multicolor fly, TIE-DYE, was designed to perform lineage tracing of 7 distinct cell populations marked in red, green, blue, yellow, teal, purple or white, and genetic manipulation of up to 4 of these populations [83]. TIE-DYE is based on a Flp/FRT mediated recombination, 3 independent constructs activating the expression of GFPNLS, lacZNLS or GAL4 and a UAS-his2A::RFP construct to visualize GAL4-expressing cells. The TIE-DYE system was used to estimate the number of founder cells giving rise to the wing-imaginal disc during normal development and to track their growth, patterning and cell–cell affinity following compensatory growth caused by X-ray irradiation [83].

Based on single transgenic constructs, a sixth multicolor fly named Raeppli was later developed to mark different cellular compartments [84]. Constructs used in the Raeppli method contain sequences of 4 distinct FP followed by stop codons (TagBFP, mTFP1, mOrange (or E2-Orange for nuclear constructs) and mKate2) that can be activated by heat shock-flippase-integrase activity and be used in combination to increase the number of hues. In a first construct, the FP are targeted to the nucleus by a nuclear localization signal to study clonal position and growth characteristics. In the second construct, the FP are targeted to membranes by a Ras farnesylation sequence to analyze cell and clone shape changes. Raeppli allows whole-tissue labeling where the descendants of the majority of cells in a single organ are labeled and can be followed simultaneously. Raeppli was validated by inducing multicolor landmarks at various stages of the wing imaginal disc development to understand its global growth characteristics and follow its prepupal eversion by performing live imaging.

A seventh approach, the MultiColor FlpOut system [85] relies on three differently tagged reporters (HA, FLAG and V5) under UAS control, kept silent by a transcriptional terminator flanked by two FRT sites (FRT-stop-FRT) and activated by a heat shock-inducible Flp recombinase. Expression of single or combined labels for a greater color panel occurs only in cells that also express a GAL4 driver. This technique was combined with specific neuronal or glial GAL4 drivers to study the adult fly visual system and later to visualize different glial subtype morphologies in the adult fly brain. It enabled to study at high resolution single cell morphologies and interactions [86].

The eighth strategy, nBitbow, was developed by randomly assigning 5 distinct, bright and stable FP whose expression can be independently switched ON or OFF upon Flp recombination, leading to up to 31 colors using a single cassette [87]. The authors also developed statistical tools to quantify the likelihood of lineage relationships between two given nBitbow-labeled cells and applied them to describe the lineage of Drosophila neurons in the peripheral nervous system.

Finally, CLADES (for cell lineage access driven by an edition sequence) is based on a CRISPR/Cas9 machinery and relies on successive genetic changes to switch ON and OFF fluorescent reporter genes in a predetermined cascade via single-strand annealing mechanism of DNA repair [88]. This system was applied to Drosophila neuroblasts lineage reconstruction in which CLADES 1.0 used two reporters (YFP, RFP) with Cas9 under the Dpn neuroblast promoter to generate CLADES-labeled neurons with three colors (Green, Red, Yellow), and where CLADES 2.0 combines a five-color cascade using 3 reporters (CFP, YFP, RFP) with a Dpn-GAL4 induction. This technique enables to assess the temporal resolution of lineage development across successive cell generations which express different combination of reporters, the genetic manipulation with programmable cascades of genes, and serial biological events studies.

### IPSCs

Induced pluripotent stem cells (iPSCs) have been largely used as a tool to generate tissue, to model human development and disease, and for reliable drug screening. El Nachef et al. took advantage of the possibility to study dynamic behaviors of iPSCs at the single cell scale with multicolor markers to better understand differentiation of human cells and in particular the heterogeneity of clonally related cells at the phenotypic level [89]. They generated a rainbow cell reporter WTC11 human iPSC line by co-electroporating a Brainbow-3.2 construct where the Thy1 promoter is replaced with a CAG promoter and which is flanked with AAVS1 homology arms for a targeting knock-in, a plasmid expressing Cas9 and a guide RNA targeting the AAVS1 locus. Cre induction led to multiple unique membrane-targeted fluorescent barcodes enabling clonal expansion and migration as well as morphological changes, revealing heterogeneous dynamics at the clonal level.

## Multiclonal analysis findings in the mouse brain and other tissues

Understanding how ontogenetically related cells contribute to build a tissue is of paramount importance to apprehend the cellular and molecular mechanisms that govern its construction. In the early days of lineage tracing in central nervous system in particular, it was successfully achieved at the single progenitor level using clonal analysis based on low-titer retroviral vector injection, carrying out reporter gene such as LacZ, alkaline phosphatase, luciferase or fluorescent proteins [90]. Nowadays, with the advent of lineage tracing techniques based on somatic or CRISPR/Cas9 mutations and DNA barcoding, several hundreds of clones can be resolved at single-cell resolution. As all the above lineage tracing techniques are the subject of excellent recent reviews [91–95], we will not discuss them here any further. Instead, we will describe some of the main findings obtained from high-throughput lineage tracing methods based on multi-fluorescent reporter activation, focusing on the development of the brain and several other biological systems, such as intestine, skin, vasculature, hematopoietic and immune systems.

### Mouse cerebral cortex and other brain regions

#### Neural clones

The MADM method initially allowed ubiquitous or specific cell labeling in various areas of the nervous system, thanks to different Cre mouse lines (Nestin-Cre, Foxg1-Cre, Hprt-Cre, Actin-Cre, Wnt1-Cre, En1-Cre), or in epidermal tissue with the keratin5-Cre line [4]. With the sparse labeling obtained by MADM6 line and the use of an inducible Actin-Cre^ER^ line, a correlation between lineage and axonal projection pattern in cerebellar granule cells has been reported [4], as well as a better understanding of their neurogenesis and differentiation timing [96]. Since its beginnings, MADM has been broadly used for neuronal lineage tracing studies, as illustrated in a lineage study of radial glia-like precursors performed in the adult dentate gyrus using MADM6 crossed with Nestin-Cre^ERT2^ mice that revealed their multipotency and led to the identification of their multiple modes of activation (asymmetric and symmetric self-renewal) [97]. MADM has also been used to investigate the regulation of cell cycle progression along with the establishment of the cell autonomous function of the zinc finger transcription factor specificity protein 2 (Sp2) in the embryonic and postnatal brain [98]. For this, Nestin-Cre mice were intercrossed with Sp2-MADM11 mice, where MADM alleles are combined with Sp2-mutant alleles on the same chromosome, allowing for genetic analysis of mutations. The authors found that Sp2-dependent mechanisms regulate cell cycle progression and neurogenesis is disrupted in its absence. By crossing the Sp2-MADM11 with FoxG1-Cre, FoxG1^IREScre^, Emx1^IREScre^ or Nestin-Cre^ERT2^ mice, they also showed that Sp2 is necessary in neural stem cells and progenitors in stage-specific manner, with mechanistic differences between the early expansive and later neurogenic corticogenesis phases [98, 99]. Additionally, the neurogenesis program of mouse neocortex was deciphered using MADM11 mice crossed with Emx1-Cre^ERT2^ or Nestin-Cre^ERT2^ mice or using the Otx1-MADM11 mutant. Relying on quantitative clonal analysis of radial glia progenitor division patterns and transition to gliogenesis program, this work revealed the deterministic behavior of radial glia progenitor division pattern [100]. In addition, the division mode of medial ganglionic eminence (MGE) progenitors and the broad dispersion of their interneuron progeny were analyzed in MADM11 mice crossed with Nestin-Cre^ERT2^ mice, showing that intermediate progenitors in MGE SVZ increase the number of post-mitotic interneurons through symmetric neurogenic divisions [101]. Finally, the formation and organization of developing thalamus were investigated in MADM11 mice crossed with Nestin-Cre^ERT2^ mice and R26^SmoM2-YFP^ mice by clonal analysis of progenitor behavior and neuronal progeny organization, showing the clonal nature of individual radial clusters and that Sonic Hedgehog signaling activity plays a role in clonal spatial distribution [102].

Moreover, MADM tools allowed the assessment of genomic imprinting impact on neural diversity and to probe imprinted gene functions during cerebral cortex development. MADM7 and MADM12 mouse lines were generated to create uniparental chromosome disomy (UPD) with somatic cells carrying two copies of either maternal or paternal chromosome. The authors analyzed the effects of preferential expression of either maternal or paternal alleles in various tissues including the brain at single cell level and revealed chromosomal and cell-type specificity of genomic imprinting effects on neural development [7]. Another study employing Cdkn1c-MADM7 and Emx1-Cre mouse lines showed that the cyclin-dependent kinase inhibitor p57KIP2 genomic locus regulates cerebral cortex development via a cell-autonomous mechanism independent of genomic imprinting by promoting radial glial cell survival [103]. Studying allelic expression of imprinted genes in principal cell types of developing cerebral cortex, Laukoter and colleagues found that, although the control of imprinting acts largely in a non-cell-type specific manner, some cell type-specific transcriptional responses can lead to specific phenotypes in response to UPD, with a special role in cortical astrocyte survival and glial astrocyte lineage [104]. In this study, the authors used MADM7, 11 and 12 mice together with Emx1-Cre or Nkx2.1-Cre mouse lines to perform transcriptional analysis of MADM-labeled FACS sorted cells.

#### Gliogenesis at clonal levels

To investigate astrocyte clonal arrangements and subtype composition during cortical development, Laura Lopez-Mascaraque’s laboratory has developed the StarTrack method that provides a fluorescent code inherited by sister cells and thereby enables the tracking of multiple clones during development. Using this in vivo long-term tracing of astrocyte lineages at clonal levels, Garcia-Marques and collaborators have shown a widespread dispersion of glial clones throughout cerebral cortex and restricted pial and fibrous astrocytic clones in corpus callosum and in cerebral cortex that suggests the existence of specialized progenitors that will contribute to generate astrocyte diversity during development [35]. Still using the StarTrack strategy, the authors also showed that the clonal behavior of olfactory bulb (OB) astrocytes differ from the cortical ones as the vast morphological diversity of OB astrocytic clones appears layer-dependent rather than determined by lineage, with astrocyte clones comprised of a very limited number of cells and arranged following a radial pattern [37]. The authors have used a similar strategy to explore NG2 cell developmental properties at clonal level and showed that this heterogeneous population is composed of large clones (in the range of several dozens to several hundred cells per clone), that increase with age and occupy several layers in adult cerebral cortex and olfactory bulb [36, 38], a clonal size range that differs from the neuronal and astrocytic populations of the cerebral cortex [46, 100]. Obtained from the tracking of several clones simultaneously, these results highlight the clonal heterogeneity of glial cells across distinct glial cell populations (astrocytes, NG2 cells) and in distinct brain areas (cerebral cortex and olfactory bulb).

Clavreul and colleagues employed large volume 3D serial blockface multiphoton imaging and confocal imaging of serial sections labeled with MAGIC Markers to perform multiclonal lineage tracing of astrocytic descent of cortical progenitors targeted by electroporation during embryonic or postnatal development [46]. They showed that the astroglial network arises from both embryonic and postnatal ventricular progenitors whose descent gives rise to scattered protoplasmic astrocytes as well as pial astrocytes. Furthermore, neighboring cortical astrocyte clones are intermixed and present high heterogeneity of size, subtype composition, spatial organization and three-dimensional dispersion. Astrocyte clones undergo a dynamic phase of dispersion and proliferation during the first postnatal week, followed by a maturation phase where individual cells continue to acquire their complex morphology [46]. This MAGIC Markers-based multiclonal analysis uncovered the tremendous variability of cortical astrocytes which strongly differs from the stereotypy exhibited by cortical pyramidal neurons during development while providing a comprehensive view of the cellular mechanisms governing the establishment of the astroglial 3D matrix.

Far from being restricted to neurons, MADM has also been applied to better understand glia development, particularly the role played by the cortical cytoskeleton protein LLGL Scribble Cell Polarity Complex Component 1 (Lgl1) in cortical astrocyte production. Beattie and collaborators used Lgl1-MADM11 mice, whose MADM alleles are combined with Lgl1-mutant alleles on the same chromosome, allowing for genetic analysis of mutations. These Lgl1-MADM11 mice were crossed with Emx1-Cre or Emx1-Cre^ER^ mice, in a WT background to obtain a sparse genetic disruption or in a Lgl1-KO background for a complete Lgl1 ablation, in order to study radial glia progenitor behavior in the developing cerebral cortex [105]. Their results suggest that Lgl1 controls astrocyte production in a cell-autonomous manner. Moreover, with a double Lgl1/Egfr mutation identified by MADM11 labeling, they showed the genetic interaction between Lgl1 and Egfr and their functional cooperation in cortical astrocyte production. MADM has also been applied to the lineage tracing of radial glial cells which showed a common lineage between ependymal cells and astrocytes and the role of Geminin family members in balancing the two lineages using in utero electroporation of Cre recombinase and antagonist regulators of GemC1 and Geminin in MADM11 mice [106]. Intercrossing Egfr-MADM11 and Nestin-Cre^ERT2^ mice allowed for clonal analysis of gliogenesis in the cerebral cortex and demonstrated that epidermal growth factor plays a cell-autonomous role in glial expansion in a non-deterministic manner [107].

Tay and colleagues combined a multicolor fate mapping system based on the Confetti reporter and computational analysis to unravel the dynamic network organization of microglia in vivo during health, disease and recovery. To unambiguously discriminate between different microglia subsets with a sufficient spatial resolution, adult Confetti; Cx3cr1Cre^ERT^ (Microfetti) females were administered once with tamoxifen and analyzed beyond the lifetime of co-labeled peripheral immune cells [108]. In healthy central nervous system (CNS), the microglial network is stable and self-renewal occurs randomly with a turn-over rate and density specific of each CNS region. In contrast, acute and focal neurodegeneration such as facial nerve transection leads to selective clonal microgliosis characterized by the emergence of daughter cell clusters. During recovery, the microglial network homeostasis is restored by both microglia emigration from healed lesion and local apoptosis [108].

All these multicolor-based multiclonal analysis have provided key findings regarding the unsuspected heterogeneity of various neural cell types. They have contributed to highlight the complexity of the cellular mechanisms responsible for the proper formation of the brain at the level of individual cell types. Moreover, new techniques based on the combination CRISPR/Cas9 genome editing and electroporation in utero now open the prospect of performing homozygous knock-in of dual fluorescent marker genes into the gene and the progenitors of interest in the developing mammalian cortex not only in mice, but also, for example, in ferrets [109]. Therefore, these works open new areas of investigation regarding the extent of cell diversity, its regulation and its possible impairment in neural disorders.

### Multicolor clonal analysis in other tissues

#### Neural crest

The Confetti approach helped to close a long-standing debate about the potency of single neural crest cells [21]. Specific to vertebrates, neural crest is a transient population of stem/progenitor cells that migrate and differentiate into a myriad of lineages as diverse as peripheral neurons and glia, melanocytes or corneal keratinocytes throughout embryogenesis and sometimes adulthood. Unlike their CNS counterparts, it remained unclear if neural crest was a homogeneous population of multipotent progenitors and/or a heterogeneous population of lineage restricted progenitors. Multipotent neural crest stem cells are characterized by the generation of daughter cells that colonize different anatomical tissues and adopt distinct fates. To explore this issue, Sommer and colleagues traced in vivo single premigratory and migrating neural crest cells expressing rare color combinations by using, respectively Confetti; Wnt1-Cre^ERT^ and Confetti; Sox10-Cre^ERT2^ mice in conjunction with differentiation markers. The study showed multipotency of the vast majority of premigratory and, unexpectedly, migratory neural crest cells of the trunk in mammalian embryo less than 2 days after tamoxifen induction, revealing that the neural crest is composed of multipotent progenitors during both premigratory and migratory phases [21].

#### Intestine

Multicolor clonal analysis has not been limited to the nervous system. To function properly, each organism must ensure optimal control of its homeostasis that relies on tight control of clonal maintenance. This is particularly crucial for tissues in constant renewal that must continuously maintain healthy function despite the constraints of continuous production of new cells and the elimination of cells that have become defective. The intestinal epithelium is a good example of a tissue with these features. This highly stereotyped structure is made up of multiple identical units of several crypts at the base of a villus. Its self-renewal is the fastest of all mammalian tissues (< 1 week) and involves migration towards the apex of the villus by differentiated cells generated from intestinal stem cells (ISC) located in the crypt at the base of each villus. Each crypt-villus constitutes a functional unit independent of its neighbors, making this structure particularly interesting for understanding at a multicellular scale how the progeny of a limited number of stem cells participate in the maintenance of homeostasis, i.e., in generating the appropriate number of differentiated cells at a given time and location. The absence of cell migration between 2 crypt-villus units allows exploration of the contribution of stem cells to the development of functional villus using a limited number of colors, as obtained with the multicolor Confetti strategy. Based on its previous work identifying Lgr5 (G protein-coupled receptor) as a marker of ISC, Hans Clevers’ laboratory has explored the lineage of these cells by crossing Confetti mice with mouse lines expressing the inducible form of the Cre recombinase for targeted induction in stem cells of interest at a given stage. The generation of this Confetti mouse line enabled short and long term tracing of stem cells in the intestinal crypt after crossing with Lgr5-EGFP-Ires-Cre^ERT2^ mice to trigger the multicolor recombination specifically in the Lgr5 + crypt base columnar cells or less specifically with Ah-Cre mice [3]. This strategy was used to demonstrate how homeostatic self-renewal of intestinal stem cells is controlled as well as the behavior of their resulting clones in healthy mice [3] or, in a later study, in a mouse model of intestinal adenomas [26]. In Apc-mutant adenomas, Lgr5 + cells generate additional Lgr5 + cells but also other adenoma cell types, such as adenoma Paneth cells. Using a similar strategy with the Rosa26-Rainbow [14] instead of the Confetti mouse, Yanai and colleagues demonstrated that Bmi1 + or Lgr5 + tumorigenic cells clonally expanded in proliferating tumors and also that Lgr5 + cells function as cells-of-origin for intestinal tumors [19].

#### Skin

As illustrated with the intestine section above, multicolor transgenic mice are ideal tools to characterize clonal dynamics at the basis of tissue homeostasis. The clonal dynamics of skin tissue homeostasis has been elegantly demonstrated by Kiarash Khosrotehrani’s laboratory which has generated “Skinbow” mice based on the crossing of Rainbow3 mice (CAG-Brainbow-1.0; [15]) with Krt14-Cre^ER^ that enables either high or low-density multicolor lineage tracing in skin keratinocytes. Roy and collaborators used these mice to investigate the cellular mechanisms of interfollicular epidermal (IFE) maintenance and to determine whether progenitors in hair follicle compartments function hierarchically. In this study, the quantitative measures of clone size evolution revealed two modes of clonal progression in the IFE: fast growing clones attached to hair follicles that increase in size during the growth phase of hair follicles, and a majority of clones that are distant from hair follicles, small in size and slow cycling, that can be mobilized by a proliferative stimulus. This study combined multicolor lineage tracing to track individual keratinocytes in the epidermis of mice that can be declined in either sparse or dense labeling, associated to modeling. The authors were able to address clonal dynamics of epidermal cells over time in the dorsal IFE while taking into account potentially rare progenitor population, which is a powerful asset of multiclonal analysis based on multicolor transgenic mice [16]. More recently, Roy and colleagues used again Skinbow multicolor fate mapping to investigate the impact of ultraviolet (UV) irradiation on the initial steps of skin carcinogenesis follicles [110]. They showed that, following UVB exposure, patches of labeled keratinocytes proliferate differentially according to their location near or away from hair. This demonstrates the usefulness of Skinbow multicolor tool to elucidate cellular mechanisms underlying skin physiological and pathological processes.

#### Vascular system

Multicolor tools have been used for studying the vasculature as well. To decipher if a second somatic mutation in individual endothelial cells is sufficient to develop cerebral vascular malformations, Detter and colleagues generated a Confetti; PDGFb-Cre^ERT2^; Ccm3^fl/KO^ mouse model. Following tamoxifen administration, this inducible CCM3 model exhibits an additional somatic mutation along with a multicolor tag in endothelial cells after the deletion of the remaining floxed Ccm3 allele as well as the rearrangement of Confetti allele. Recombination occurs at both Ccm3 and Rosa26 loci within individual- colored endothelial cells that also exhibit increased expression of an indirect marker characteristic of the Ccm3 loss. Clonal expansion of colored mutant endothelial cells has been observed at different stages of the cerebral cavernous malformation, suggesting that a single somatic mutation suffices to trigger this pathology. Nearest neighbor analysis of their distribution also revealed that clonal dominance is a characteristic feature of vascular malformation. The vascular malformation content evolves from being almost exclusively composed of clonally dominant mutant endothelial cells in small cavern to a mixed population containing both a single mutant clone and unlabeled putative wild type endothelial cells [111].

#### Hematopoietic system

Another field in which multicolor fate mapping has been shown a useful tool is the hematopoietic system. The clonal contribution of RGB-transduced mouse hematopoietic stem cells (HSC) and hematopoietic progenitors to the establishment of colony-forming unit in the spleen of irradiated mice was analyzed 10 days after the transplantation [51]. Most of the spleen colonies are homogeneously colored, suggesting a monoclonal origin and expansion [51]. To further define the molecular basis of HSC heterogeneity, Yu and collaborators overlaid the in vivo functional behavior of endogenous HSC clones with their gene expression and epigenetic characteristics at a clonal level using endogenous fluorescent tagging [112]. For this purpose, the authors generated a novel multi-fluorescent “HUe” mouse strain based on Brainbow-2.0 strategy. It relies on four pairs of lox sites flanking 4 FP that enable Cre excision and inversion schemes resembling the Confetti mouse design with around 20 tandemly integrated cassettes offering a wider range (theoretically > 10^3^ color combinations). This multi-fluorescent mouse model enables both molecular profiling and functional tracking of live cells in vivo. Crossed with interferon inducible Mx1-Cre mice with injection of plpC to activate endogenous hematopoietic cells, single clusters of cells with immunophenotypic signature of one type of progenitor were sorted then transplanted into sub-lethally irradiated mice whose spleen is harvested at E11, and DNA fingerprint performed. This methodology enables the assessment of HSC-endogenous clonal behavior that can be quantitatively monitored in vivo under varying conditions, such as inflammatory or genotoxic conditions, concurrently to their transcriptional and epigenetic states. Interestingly, individual clones do not change in behavior after transplantation in terms of cell proliferation (defined by clone size) and lineage commitment, and the epigenome provides lineage-constraining boundaries within which lineage bias will eventually be resolved. Therefore, this study challenges the current line of thought that stem/progenitors are relatively plastic cells that can respond variably to their specific environment. By demonstrating that HSC clones display an unanticipated stereotypical behavior upon transplantation, this work shows that the wide range of HSC clonal behaviors is highly constrained by cell intrinsic features such as DNA methylation and chromatin accessibility [112].

#### Immune system

The multicolor strategy based on the Cre/lox recombination system represents a powerful tool to understand the dynamic cellular behaviors of immune system cells [17]. Indeed, multicolor fate mapping based on Brainbow-1.0L strategy has been successfully applied to investigate the homeostasis of resident immune cells, such as Langerhans Cells (LC) and dendritic epidermal T cells (DETC). In an initial study, Marc Bajénoff’s laboratory generated a new transgenic mouse line based on Brainbow-1.0L construct under the control of strong human Ubiquitin C promoter (Ubow mouse) to investigate the cellular mechanisms that regulate the homeostatic renewal of the LC network and its replenishment after inflammation [17]. LC are long-lived dendritic cells that reside in the skin and migrate continuously from the epidermis to the lymph nodes (LN). This migration of LC is a priori compensated by a local proliferation of mature LC, but this hypothesis had not been experimentally tested so far. Intercrossing Ubow mice with a mouse line expressing Cre recombinase in LC (Lang-Cre), Ghigo and colleagues demonstrated the presence of LC groups consisting of a dividing LC and its daughter cells, all sharing the same color. These single-colored groups invalidate the hypothesis of replacement of emigrating LC by the division of one of their closest neighbors, which would have resulted in a random distribution of cells of the same color. Indeed, if this hypothesis was validated, the location left empty by the emigrating LC being filled by the division of one of its neighbors at random would have generated a stochastic distribution pattern of colored cells [17]. Although skin-derived CFP + and YFP + LC were randomly distributed in the draining LN, epidermal CFP + and YFP + LC were not randomly distributed in the epidermal skin but assembled in monocolored foci. This work shows that the adult LC network is constituted by contiguous proliferative units composed of tissue-resident dividing LC and their daughter cells. In addition, a few immature LC are in charge of replacing neighboring emigrating LC and thus responsible for the maintenance of proliferative units [17]. Using a similar strategy based on intercrossing Ubow with specific Cre mouse lines (Ubow;Cx3cr1-Cre fate mapping strategy) where recombination occurs in lymphoid DETC, the hematopoietic origin of this other type of epidermal resident immune cells was explored. This strategy helped to understand the cellular mechanisms regulating DETC homeostasis, which relies on a network composed of adjacent proliferative units in the adult epidermis and homeostatic maintenance of adult DETC through clonal proliferation [113]. Intercrossing of Ubow multicolor reporter mice and specific Cre lines such as Wnt1-Cre (neural crest cell derivatives), CD21-Cre to label two subsets of stromal cells: follicular dendritic cells (FDC) and versatile stromal cells, and RAG-2^o/o^ (all stromal cells), allowed to dissect the origin and dynamics of lymph node follicular dendritic cells. This study offers new insights into the cellular mechanisms driving the development and remodeling of LN FDC. These cells arise from the proliferation of tissue-resident progenitors of mesenchymal origin, and the accumulation of FDC in reactive LN does not rely on the recruitment or the proliferation of FDC [114]. In addition, this multicolor lineage tracing approach unraveled the first and pivotal biological function of marginal reticular cells as progenitors of LN FDC during B cell follicle development and remodeling [114]. Beyond a better understanding of tissue homeostasis maintenance, Ubow multicolor strategy was also used to investigate cellular mechanisms involved in LN vasculature remodeling upon immune response, as it enables tracking the behavior of blood endothelial cells (EC) during LN expansion upon inflammation and subsequent return to homeostasis. LN vasculature is composed of capillary blood EC and high endothelial venule (HEV) cells. During an immune response, LN expansion relies on the transient remodeling of its vasculature. Imaging of the vasculature network of the adult mouse LN at single-cell resolution using Ubow;Cdh5-Cre^ERT2^ mouse model (in which the tamoxifen-inducible Cre recombinase is placed under the regulation of vascular endothelial cadherin promoter) enabled to track the individual behavior of LN EC during the inflammatory response and its resolution. Using this multicolor strategy, Mondor and collaborators showed that EC clonally proliferated and sequentially assembled into contiguous segments along the vascular tree of LN. HEV cells acted as local endothelial progenitor cells to create capillaries and HEV neo-vessels at the periphery of LN. After the peak of the immune response, the return to homeostasis was accompanied by the stochastic death of pre-existing and neo-synthesized LN EC [115].

In addition, several studies carried out within the same tissues (skin and intestine) of multicolored mice explored in more detail the action of Wnt/β-catenin [116] and K-ras [117] pathways in a context of clonal competition. Although using distinct multicolor mouse lines and focusing on different tissues in physiological or pathological conditions, all these studies illustrate the value of using color-based multiclonal analyses to resolve the complex mechanisms elaborated by tissues at the multicellular scale to maintain their homeostasis.

## Contribution of multicolor strategies to the study of tissue morphogenesis and homeostasis

### Potentialities of neural progenitors at population level

Two topics particularly debated in developmental neurobiology concern the homogeneity of embryonic stem cell potentialities and the extent of their contribution to the pool of adult neural stem cells (NSC) that continues to generate neurons in the mature brain.

#### Zebrafish CNS

Dirian and collaborators have used multicolor tracing approaches to decipher the embryonic origin of NSC in zebrafish. They have thus shown the participation of two spatially and molecularly distinct populations of embryonic stem cells in the adult neural stem cell population: one sensitive to the Notch (Her4+) pathway located in the germinal region of the pallium generates dorsomedial adult neural stem cells; while the other, independent of the Notch (Her4−) pathway, is constituted by progenitors adjacent to the telencephalon roof and generates adult neural stem cells from the lateral pallium. Surprisingly, these two populations remain segregated throughout brain development and participate asynchronously in neurogenesis: the Her4+ progenitors participate in production of neurons from early embryonic stages while the Her4− population contributes essentially to late phases of neurogenesis [118].

#### Mouse forebrain and cerebellum

These fundamental questions regarding the embryonic origin of adult NSC addressed in zebrafish are still partially unanswered in mice. Indeed, the central nervous system of mammals, and in particular the cerebral cortex, presents properties that make it very difficult to explore these subjects, such as the location of embryonic stem cells deep in the tissue, close to the ventricles, and the absence of markers that allow their individualization. In addition, the large amplitude of dispersion of the progeny of these stem cells during cerebral development requires a larger number of stable and easily distinguishable markers in order to link sister cells together with the smallest margin of uncertainty. These constraints were poorly compatible with the limited color palette offered by multicolored transgenic mice available thus far. Therefore, acute transgenesis relying on the integration of multiFP reporter constructs under the control of a ubiquitous promoter has been developed to track the entire progeny of neural progenitors, regardless of the cellular identity adopted by the daughter cells (i.e. neuronal, astrocytic, oligodendroglial) with an extended palette of color combinations. MAGIC Markers (MM) strategy based on Brainbow transgenes provides a wide range of markers to individualize a large number of neighboring cells. Following in utero electroporation of MM transgenes at a precise developmental stage where the combinatorial markers are inserted in the genome of dividing cortical stem cells, the persistence of color combinations over numerous rounds of cell divisions enables to track cell identity and location of sister cells during brain development [119]. This strategy showed that dividing cortical stem cells during the early stages of embryonic neurogenesis contributed to the pool of adult NSC that generated the neurons of the olfactory bulb during adult neurogenesis [1] and that cortical astrocyte development is plastic at clonal and cellular levels [46]. However, recapitulating the entire progeny of progenitors including neuronal and glial lineages from embryonic to adult stages using MM strategy is hindered by the expression of FP from the episomal plasmids at the time of electroporation prior to their integration in the host progenitor genome. As a result, neurons born at the time of electroporation express a wider palette of colors resulting from the expression of FP from both integrated and episomal vectors. This limitation has been overcome recently by Kumamoto and collaborators by developing an iOn strategy that permits FP expression solely upon integration of the reporter into the host cell genome, and prevents FP expression from episomal constructs [48]. Prior to iOn, Figueres-Onate and collaborators had developed another ubiquitous lineage tracing method based on a combination of the Cre/lox recombination system and StarTrack constructs whose FP are driven by a ubiquitous UbC promoter. Upon tamoxifen injection, the inducible Cre recombinase cleaves the FP genes flanked by the loxP sites in the episomal constructs that have not been integrated into the electroporated progenitor genome [40], thus preventing FP expression outside of integrated copies. Using this method, the authors provided a qualitative representation of diversity exhibited by clones encompassing neuronal and glial lineages that remains to be assessed in a quantitative manner, which thus far has only been possible using the MADM strategy. In addition, Figueres-Onate and collaborators explored the precise relationship between embryonic and postnatal neural progenitors and their commitment to neuronal and glial lineage using an electroporation-based genetic lineage tracing performed at either embryonic or postnatal stages and using a combination of integrative eGFP and non-integrative mCherry constructs. This study confirmed that neuronal lineages arise from many progenitors in the proliferative zone after few cell divisions, whereas glial cells arise from fewer progenitors undergoing fewer cell divisions [120]. The heterogeneity and potentialities of progenitors have also been investigated in the cerebellum, where Parmigiani and collaborators have used a combination of in vitro lentiviral infection, ex vivo StarTrack and in vivo R26R-Confetti;GLAST-Cre^ERT2^ multicolor clonal analysis to provide evidence that the postnatal prospective white matter hosts a bipotent progenitor with astroglial features that gives rise to both interneurons and white matter astrocytes [121]. Following this work, Cerrato and collaborators have delved further into cerebellar astrocyte development. Using StarTrack-based clonal analysis, proliferation studies and computational modeling, the authors have shown that the generation of astrocytes follows a well-defined spatiotemporal pattern and relies on precursors whose fate potential declines over time [122].

#### Mouse adult germinative zones in olfactory bulb and hippocampus

The lentiviral multicolor RGB labeling approach has been also applied to track the fate of adult NSC using respectively the CMV and SFFV promoters with the same VSVG (vesicular stomatitis virus glycoprotein) envelope [52]. The promoter choice impacts the type of cell that will be marked 6 weeks after the intraventricular administration. Lentiviral-transduced subventricular NSC have been described to produce only granular neurons in the olfactory bulb with the CMV promoter and striatal glia cells with the SFFV promoter. The origin of these newly generated glial cells is likely gliogenic as they are only labeled after intraventricular and not striatal injection of VSVG-SFFV lentiviral vectors. RGB marking was further applied to illustrate the temporal dynamics of neurogenesis in the hippocampus. Gomez-Nicola and colleagues took advantage of the aptitude of MLV-derived γ-retroviral vectors to transduce only dividing cells and improve the transduction efficiency towards NSC by using mouse ecotropic envelope. Age-related increase in dendritic spine density has been shown to reflect progressive maturation of newly generated granule cells using sequential hilus injection of Venus and mCherry Eco-SFFV retroviral vectors [52].

To understand the age-dependent decline in neurogenesis, NSC dynamics have been studied at single-clone resolution in young adults [24] and middle-aged mice [29]. The progeny of individual NSC located in the SVZ were tracked in vivo along the rostral migratory stream in sparsely labeled Confetti; Glast-Cre^ERT2^ mice [24, 29]. Throughout adulthood, fast clonal expansion of single labeled NSC generates a progressive wave of new interneurons in the olfactory bulb. Exclusively neurogenic, the majority of clones are only composed of deep granular neurons, while a minority consists of both deep and superficial granular neurons. However, the long term self-renewing capacity of most NSC appears already limited in young adults as clones containing both NSC and terminally differentiated NSC progeny were rarely detected a few weeks after the labeling induction [24]. By applying a mathematical model representing the temporal evolution of the abundance of different cell types observed per clone in young and middle-aged animals, Bast and colleagues nevertheless identified subtle clonal dynamic changes with aging. Increased probability of NSC asymmetrical division generating one NSC daughter along with one differentiated daughter and extended quiescence are both predicted to participate in the adult neurogenesis decline [29].

### Stem cell contribution to tissue morphogenesis outside of the brain

#### Cardiogenesis

Multicolor strategies are essential when it comes to tracing the fate of multiple cells individually. Each new multicolor strategy helps to determine how multiple cells participate in the development of a cellular mechanism or biological function. With respect to tissue formation during development, multicolor strategies are critical for detailing individual contributions of stem cells to the morphogenesis of a tissue/organ, as elegantly demonstrated by Gupta and Poss regarding the cellular mechanisms responsible for cardiac development in zebrafish [70]. They generated a multicolor zebrafish line under the control of the beta actin 2 promoter (called priZm) which, when crossed with a line restricting the expression of the inducible form of recombinase Cre to cardiomyocytes (Cmlc2-Cre^ER^), allows mosaic recombination only in the presence of 4-hydroxytamoxifen (4-OHT). By analyzing the size, shape and surface area occupied by cardiomyocyte clones from embryonic to adult stages, Gupta and Poss showed that (i) the ventricular wall of juvenile zebrafish (30 days post-fertilization) is formed from the lateral expansion of several dozen cardiomyocytes, (ii) during the subsequent maturation stage, the juvenile heart is progressively enveloped by a new lineage of cortical muscle, (iii) the adult cortical muscle (> 3 months post-fertilization) is formed from a small number of cardiomyocytes (on average 8 per animal) which displays a clonal dominance over time, reminiscent of the stem cell population dynamics presented by the ISC of the intestinal epithelium [3]. While providing information on the high degree of clonal heterogeneity in terms of size, shape and surface area occupied between animals, this multiclonal analysis identified a common developmental process and two distinct types of ventricular myocardium that had previously gone unnoticed [70]. Multicolor tracing approaches permit to simultaneously follow a large number of cells and thus allow exploration of the contributions of different progenitors to the genesis of an entire organ. By rapidly extracting the main cellular mechanism that follows the development of this structure and visualizing the degree of variability between animals, it is now possible to identify “rare” phenomena that co-exist with the principal cellular mechanism. Thus, the study of clonal patterning of embryonic cardiomyocytes shows that they generally remain connected to each other within the same clone. However, in a systematic way, about 10% of clones show a complete separation of cardiomyocytes from their clonal partners (or sister cells). These multicolor tracing approaches thus provide a large amount of information on the majority and minority cellular mechanisms governing tissue morphogenesis while highlighting the high degree of intra- and inter-animal heterogeneity.

#### Early embryogenesis

Like Gupta and Poss, other teams are exploring the individual contribution of stem cells to the formation of different tissues in zebrafish [61] and mice [15]. For example, Tabansky and colleagues used the Brainbow-1.0 strategy with a ubiquitous promoter (Rainbow, Fig. 1) to determine whether blastomeres at the 4-cell embryonic development stage (or cleavage stage) contribute identically or differently to the generation of internal cell mass (ICM, future embryo) and trophectoderm (TE, future placenta). By inducing recombination at the 4-cell stage and analyzing the distribution of labeled clones within the ICM and TE, the authors suggest a biased contribution of blastomeres to these two structures, while underlining the absence of causality between this bias and the animal hemisphere/vegetative hemisphere localization of the clones [15].

#### Adult mammary gland

In another multicolor model, Visvader and colleagues used the R26-Confetti mouse to demonstrate in vivo the existence of bipotent stem cells in murine adult mammary gland [123]. Focusing on tracing the lineage of keratin 5 (Krt5)-expressing cells and their contribution to ductal homeostasis, the authors analyzed over time the composition and size of the labeled clones located along the mammary ducts in Krt5-rtTA;TetO-cre;R26R-Confetti mice having received a single injection of the antibiotic doxycycline (necessary for the activation of Cre recombinase) one to several weeks prior analysis. They demonstrated the existence of bipotent basal stem cells that generate both myoepithelial and luminal cells, having a long lifespan and a strong capacity for expansion and actively participating in ductal homeostasis throughout the life of the animal [123].

#### Corneal endothelium

Beyond their critical role in multiclonal analyses and genetic fate mapping, multicolor transgenic mice give access to complex 3D cellular morphologies and are valuable tools to determine the cell autonomous effects of signaling pathways during development. To illustrate, MADM mice helped to visualize the 3D shape of individual endothelial cells within the native monolayer of corneal endothelium during development and showed that endothelial cells possess an elaborate multipolar shape far from being simple polygonal prisms. Individual cells display a substantial heterogeneity in the number and ramifications of their lateral cell extensions. This unexpected complexity of the tissue has been revealed by the distribution of color markers in neighboring endothelial cells offered by crossing MADM mice with HPRT-Cre mice [124]. In addition to probing neighboring cell complex morphologies and interdigitations, the MADM technique allows the investigation of gene function at the level of individual cells. Aiming at dissecting in mouse corneal endothelium the role of p27, which is a critical factor regulating cell cycle withdrawal in many lineages of developing organisms, Defoe and collaborators used a combination of MADM and two loss-of-function mice. A full loss-of-function knockout line and a partial loss-of-function knock-in strain prevent interaction of p27 with cyclins and cyclin dependent kinases but leave other functions intact. MADM contributed to sporadic induction of homozygous mutant and WT cells in heterozygous endothelial monolayers. This work showed that p27 influences endothelial cell division through both cell cycle-dependent and -independent mechanisms, and has opposite effects on cell dispersion within endothelial monolayers, which confirmed previous work documenting p27 involvement in cell migration [125].

## Delving into brain cytoarchitecture using multicolor approaches

### Mapping of neural circuitry

Beyond clonal analysis, multicolor labeling is a powerful tool to study neural morphology, cellular interaction and network architecture.

Mapping the intricate neuronal and glial networks is the first step to understand brain function. Neuronal morphology reflects its connectivity potential but the tracing of their thin processes, long-range axonal projections and complex dendritic arborization is challenging at the cellular level. Brainbow models offer a wide range of colors allowing to distinguish and trace many neighboring cells [2, 12]. The Brainbow-derived multicolor strategies associated with cutting-edge imaging technologies have been used to dissect out the complex neural connectome in several brain regions. In the initial Brainbow article, Livet and colleagues traced the cerebellar circuit between mossy fiber axons and their postsynaptic granule cells in the Thy1-Brainbow-1.0H; CAGGS-Cre^ERT2^ inner granular layer. They reconstructed 341 axons and 93 granule cells in a small volume of 160 µm^2^ × 65 µm and revealed that individual cerebellar granule cells are innervated by multiple mossy fibers [2].

To achieve high-resolution imaging of large opaque samples, Emmanuel Beaurepaire’s laboratory developed the chromatic multiphoton serial microscopy (ChroMS, [47]). This new method relies on automated serial block-face tissue slicing followed by single multicolor two-photon excitation of the three recombined FP present on the MAGIC Markers (MM) transgene through wavelength mixing [45]. With ChroMS, Abdeladim and colleagues acquired high-resolution images over several continuous cubic millimeters of brain tissue, which enabled them to assess astrocyte morphology and contacts in the mouse cerebral cortex after electroporation of the Cytbow MM in embryonic progenitors [46, 47]. Furthermore, they performed large tissue volume multichannel tracing of multiple layer 2/3 pyramidal neuron processes within a densely multicolor labeled environment which was obtained by in utero electroporation of the non-integrating CAG-Cytbow transgene in mouse cortical progenitors. At the organ scale, serial mosaic imaging of transgenic MM (CAG-Cytbow; Nestin-Cre) brains resulted in a multicolor 3D whole-brain dataset consisting of multiple self-registered three-channel coronal planes acquired every 100 μm from olfactory bulb to brainstem. It provided a comprehensive neuroanatomical map of labeled neurons and glial cells with individual cell resolution at the whole-organ scale where color contrasts facilitate cell distinction. In addition, the authors performed multiplexed brain-wide mapping of neuronal projections on mouse brains injected with three distinct anterograde AAV tracers (AAV-tdTomato, AAV-EYFP and AAV-mTurquoise2) in three distinct areas of the sensorimotor cortex. Using the whole-brain serial 2D mode of ChroMS combined with high-resolution continuous-mode imaging of sub-volumes within the striatum, they obtained both a global map of the labeled projections and a detailed view of their axonal components in a given region [47].

Combining expansion and light microscopies with a multicolor approach to decipher the connectome with nanometric details and over several millimeters is nowadays within reach. The technical aspects of expansion microscopy methods are discussed in more detail in the “Imaging solutions and new software tools for quantitative analysis of multicolor data sets”, while their main applications and advances are presented hereafter. ProExM has been applied to illustrate the mammalian brain circuitry at high-resolution using confocal microscopy allowing to unambiguously resolve intermingled neurites [126]. Furthermore, individual morphology and network organization of serotonergic neurons have been revealed in Bitbow-2.1; TRH-GAL4 Drosophila larvae by applying a variant of proExM [127]. In the ventral nerve cord, 21 serotoninergic neurons have been reconstructed with nTracer [128] belonging to at least 8 newly different morphological subtypes at unprecedented resolution with a confocal. In addition, ipsilateral serotonergic neurons have been described to fasciculate their neurites together into a single projecting commissure toward the contralateral side. Finally, a bilateral symmetric network was discovered: serotonergic neurons sharing a common hemi-segment have a contralateral morphological homologue. This suggests a stereotyped arrangement among a genetically defined population despite the morphological diversity [127]. MiriEx allows to directly link an interneuron subtype to its individual morphology after stereotaxic delivery of Brainbow AAVs into Parvalbumin-Cre and Somatostatin-Cre double transgenic nuclei and immunostaining [56]. In the amygdala, 4 different molecular subtypes of interneurons were thus described: parvalbumin, parvalbumin/calbindin, somatostatin and somatostatin/calbindin inhibitory neurons. Morphological reconstruction of 53 interneurons present in one brain section with nTracer has shown that somatostatin neurons seem to have a simpler dendritic arborization than parvalbumin neurons despite a similar dendritic length [56]. Independently of Brainbow neural circuit, iExM enables nanoscale imaging of the whole putative synapse by resolving even through epifluorescence pre- and postsynaptic elements from the synaptic compartment, respectively label with Homer1, Gephyrin and GluR1/ GABA_A_Rα1-α2 in cultured neurons [129]. Regional heterogeneity in both synaptic architecture (broad synaptic space in the medial pallidum versus close one in the S1 cortex and striatum) and tiling (even distribution in the cortex and striatum versus cylindrical patterning in the pallidum) has also been shown with iExM on a confocal microscope [129]. To illustrate how synaptic input map can further refine neuronal subtype classification, Shen and colleagues correlated Brainbow labeling of parvalbumin neurons with Bassoon- Gephyrin and Bassoon- Homer1 immunostaining, respectively marking putative inhibitory and excitatory synapses via confocal imaging [56]. Reconstructing the different synaptic inputs on the dendritic tree of 5 parvalbumin neurons has shown that some neuron arbors either a balanced or skewed distribution of excitatory versus inhibitory inputs [56]. By disclosing individual morphological heterogeneity among a densely marked network, multicolor nanoscale imaging reveals network pattern between genetically related serotonergic neurons in fly [127] and link morphological feature to a neuronal subtype in mice [56].

Dynamic modulation of the underlying neuronal connectome by glial cells adds another level of complexity to our understanding of the nervous system. Anatomical interactions among glia and between glia and neurons can likewise be unambiguously identified with multicolor tools. For example, terminal Schwann cells, Bergmann glia and astrocytes are labeled in several Thy1- Brainbow lines (B, G, M and Q, [2]). Singled out after electroporation of MAGIC Markers in dorsal progenitors, individual cortical astrocytes progressively increase their volume and process complexity whereas clonal expansion and proliferation stops between the first and third postnatal week [46, 119]. Furthermore, adult neighboring astrocytes marked with membrane-targeted FP display a continuously tiling of the cortex while interdigitating in the colliculus of Thy1-Brainbow1.1 line M; CAGGS-Cre^ERT2^ mouse [2]. Preferential coupling among sibling astrocytes has been shown after intracellular dye injection in StarTrack-labeled clone of astrocytes [130], although the mechanism of this preferential coupling is still unknown. The nearly entire tridimensional morphology of a cortical astrocyte is attainable with the large volume ChroMS approach [47]. Layer-specific differences in protoplasmic astrocyte morphology and putative neuronal soma engulfment within each astrocytic domain has thus been quantitatively revealed: a layer 4 astrocyte makes contact with more cells, yet its territorial volume is smaller than a layer 5a astrocyte [47]. Examples of neighboring oligodendrocytes sharing several axons to myelinate have been described using a CAGbow; PLP-Cre^ERT2^ model [20]. In Thy1-Brainbow-1.0G; CAGGS-Cre^ERT2^ cerebellum, multiple Bergman glia cells were reported to enwrap the same Purkinje cell dendritic tree [2]. As they provide stable and strong color labels, Brainbow and derivatives are compatible with in vivo imaging for longitudinal studies [1, 2]. Dynamic boundary rearrangement between adjacent terminal Schwann cells ensheathing a single motor axon have thereby been imaged over a timespan of 50 days at the Thy1-Brainbow-1.0 line B; CAGGS-Cre^ERT2^ neuromuscular junction [2].

### Neuronal migration and morphogenesis

In addition to its primary use in clonal analyses, MADM has been applied to the study of neuronal migration. MADM11 was used to assess Lissencephaly-1 (Lis1) and the nuclear distribution gene E-like homolog 1 (Ndel1) cell-autonomous and cell-non-autonomous functions in cortical neuron production and migration [5]. Lis1 is involved in radial migration of neocortical projection neurons. Moreover, Ndel1 binds to Lis1 and is also essential for cortical neuron migration. Intercrossing Nestin-Cre^ER^ mice with the separate recombinant MADM^GT^ and MADM^TG^ strains for null mutants of Lis1 and Ndel1 respectively, Hippenmeyer and collaborators showed that Lis1 regulates neuronal migration efficiency and Ndel1 influences the entry into the target lamina [5]. In terms of laminar fate, the analysis of intermediate progenitor progeny using MADM11 together with the intermediate progenitor specific Tbr2-Cre^ER^ mouse line, revealed laminar fate multipotency and daughter cell apoptosis of mouse cortical intermediate progenitors [131]. In addition, the assembly of the neocortical cytoarchitecture and layering was assessed by analysis of the contribution of individual progenitors [132]. Using MADM11 and the specific Emx1-Cre^ERT2^ mouse line together with mathematical modelling, the authors demonstrated stochasticity in cortical neurogenesis leading to diverse cytoarchitectures characterizing different cortical areas.

Multicolor models have been employed in multiple independent studies to explore neuronal morphology and development at the level of individual dendrites. Dendrite morphogenesis in Purkinje cells was investigated with the functional analysis of neurotrophin receptor tropomyosin-related kinase C in mouse neural development using MADM7 together with TrkC conventional or conditional knockout and the pan neural Nestin-Cre or the Purkinje cell specific pcp2-Cre lines [133]. The authors showed that TrkC, and particularly its kinase activity, is cell-autonomously required for Purkinje cell dendritic arborization. The regulation of spine density was studied through the characterization of ephrin-B3 role in synapse number and competition in cortical neuron layers 5–6 [134]. The authors used MADM11 mice crossed to mice heterozygous for an ephrin-B3 null allele and the specific Emx1-Cre and Nestin-Cre mouse lines to show that Ephrin-B3 controls excitatory synapse density via cell–cell competition for EphB ligands. Using NR2B-MADM6 mice, where MADM alleles are combined with NR2B-mutant alleles on the same chromosome, allowing for genetic analysis of mutations, along with Nestin-Cre or Nestin-Cre^ER^ mouse lines, Espinosa and colleagues analyzed the cell-autonomous function of NMDA receptor 2B in dendrite development in mouse hippocampus. They showed that NR2B regulates dendrite patterning in a cell-autonomous manner to represent sensory information in the cortex [135]. Following a primary color mixing approach to generate multicolor hues, three γ-retroviral vectors encoding mCherry, Venus and mCerulean were created using the SFFV promoter and ecotropic envelope to trace hippocampal neurogenesis through continuous and progressive dendritic spine increase [52]. Unresolved without expansion, details as small as Brainbow-AAV-labeled dendritic spines are distinguishable yet complicated to analyze in proExM-processed hippocampal molecular layer with conventional confocal imaging [129]. After iExM, dendritic spines become easily resolved with a confocal to further analyze their individual morphological features such as the number, size or shape. Of note, less membrane-targeted FP are observed at the postsynaptic zone suggesting that the synaptic cleft prevents the diffusion membrane-bound protein toward this subcellular compartment [129].

### Angiogenesis

Pontes-Quero and colleagues used ifgMosaic to study the proliferation dynamics of high/low VEGF and high/low Notch cells during neural tube development and during retinal angiogenesis [33]. By crossing iChr-Notch-Mosaic mice with Polr2a-Cre^ERT2^ mice (RNA Polymerase II Subunit A) for ubiquitous expression, the authors observed impaired neurogenesis and reduced expansion of individual progenitor cells after inducing either low or high Notch levels in neural progenitors, despite producing different cell-differentiation outcomes. They crossed the endothelial cell-specific Tie2-Cre and Cdh5(PAC)-Cre^ERT2^ mouse lines with ifgMosaic mice. The resulting mosaic genetic manipulation of vascular endothelial growth factor receptor 2 (VEGFR2) and Notch allowed the authors to analyze the variable proliferation behavior of endothelial cells induced by different levels of these signaling factors in the retina during angiogenesis [33]. In a second study on endothelial cell development in the retina, the authors combined iChr-Notch-Mosaic, iMb-Vegfr2-Mosaic and variants of these mouse lines with pharmacological experiments to analyze the functional interactions between VEGF, Notch, ERK, and the cell cycle inhibitor p21 in angiogenic and quiescent vessels [136]. They showed that mitogenic stimulation induced by VEGF, or Notch inhibition, stopped the proliferation of angiogenic vessels, via a dose–response effect to VEGF and MAPK activity (ERK signaling) which is neutralized by Notch and p21 [136]. Finally, they mapped the expansion and arteriovenous fate of endothelial cells after inducing Notch or VEGF loss- and gain-of-function genetic mosaics by crossing ifgMosaic mice with various specific Cre mice [137]. By combining the ifgMosaic strategy with transcriptomics, the function of the VEGF and Notch signaling pathways in cell proliferation, differentiation and mobilization in the retina and the heart was redefined. This study showed that artery development relies on the timely suppression of endothelial cell-cycle progression and metabolism [137].

Moreover, using ifgMosaic tool, endothelial cell development can be studied by inducing Cre recombination in iChr‐Notch‐Mosaic ES cells and mouse zygotes to manipulate levels of the Notch pathway activity at early developmental stages. Menchero and collaborators found that Notch plays a role in coordinating exit from pluripotency and promoting cell differentiation in ES cells and is active from the 4-cell embryonic stage. Manipulating Notch levels in these zygotes was also shown to induce cells to adopt an inner or outer position at later stages [138].

The contribution of clonal expansion to vessel growth was determined with a Confetti mouse by tracking clonally expanding endothelial cells in the normal or pathophysiological retina [139]. These Confetti mice were crossed with a Cre^ERT2^ under the Cdh5 (vascular endothelial cadherin) promoter mouse line to induce and analyze specific labeling in retina endothelial cells at different postnatal stages. The obtained results indicate that most of the formed vessels do not derive from clonally expanding cells in a healthy retina whereas in a pathophysiological angiogenesis model (retinopathy of prematurity) vessel clonal expansion was sevenfold higher. Similar clonal expansion was found in a model of ischemia-induced neovascularization, after acute myocardial infarction was induced. This clonal expansion was reduced by inhibiting VEGFR2. A hind limb ischemia also led to clonal expansion, showing that VE-cadherin + cells clonally expand in the retina, the heart, and skeletal muscle after ischemia, while physiological retinal vessels grow in a randomized manner [139].

## Multicolor strategies in pathological contexts

Besides healthy organogenesis, multicolor mouse models have also proven to be very effective in the study of clonal dynamics and tissue homeostasis occurring in pathological contexts such as tumors, injuries and multiple sclerosis.

### Tumors

In addition to their major contribution to a better understanding of stem cell dynamics and their involvement in healthy tissue homeostasis, multicolor approaches help to reveal in situ the presence of previously unidentified tumor stem cells [26]. To determine whether such a cancer stem cell type exists in a well characterized model of cancer of the intestinal epithelium, the adenoma, Schepers and collaborators took advantage of the Confetti mouse to change the color expressed by cells with successive recombination triggers. A first activation of the Cre induces the expression of a color in a stem cell and its progeny, and a second activation of the recombinase carried out several days later induces the expression of a new fluorescent protein in the same cell (and its progeny). The authors have thus traced the lineage of adult stem cells from the intestinal epithelium (Lgr5 +) from which the Apc gene has been deleted. A first injection of tamoxifen in Lgr5eGFP-Ires-Cre^ERT2^;Apc^fl/fl^;R26R-Confetti mice allowed to label a mutated intestinal stem cell and to follow the contribution of its progeny to the formation of one adenoma per villus-crypt unit. Within this single-colored adenoma, a second trigger of the Cre enzyme induces the color change of a Lgr5 + cell whose Apc gene is inactivated. By showing that this cell presenting a new color is localized within the unicolor adenoma and continues to proliferate while generating all the cell types found in an intestinal adenoma, Schepers and colleagues thus elegantly demonstrated the existence of cancerous stem cells within this intestinal tumor model [26].

Multicolor tools have been successfully used to study mouse models of pathologies, and notably medulloblastomas [140] and gliomas [141], the heterogeneity of which in terms of cellular composition and proliferation has been largely described but is still poorly understood. MADM11 was used to perform lineage tracing in a mouse model of glioma after a double p53/Nf1 mutation was induced in NSC and identified by MADM labeling. Crossing this model with Nestin-Cre or hGFAP-Cre mice for NSC specificity or with NG2-Cre mice for oligodendrocyte progenitor cells (OPC) specificity revealed, with additional proliferation makers, OPC as cancer cell-of-origin [141]. In these mutants, MADM alleles are combined with the tumor suppressor proteins p53 or Neurofibromin 1 (Nf1) mutant alleles on the same chromosome, allowing for genetic analysis of mutations. The OPC as cancer cell-of-origin were confirmed by the induction of the mutation directly in OPC, leading to gliomagenesis [141]. With a different approach, HRasV12 and AKT oncogenes were introduced into radial glial progenitors to induce tumor formation by in utero electroporation in E14-E15 rat embryos. An integrated multicolor clonal labeling was triggered by the coelectroporation of a helper plasmid expressing the PB transposase under the GLAST promoter and a combination of donor plasmids containing eGFP, mRFP, or CFP transgenes flanked by PB transposons under a CAG promoter [142]. A large tumor had developed within 3 weeks, composed of both mono- and multicolor areas, illustrating not only single clonal expansion, but also clonal mixing. Moreover, electroporation of these oncogenes under the control of CAG, myelin basic protein (MBP), or GFAP showed that oncogenic events occurring in different cell types and molecular environments lead to different kinds of tumors. Another strategy took advantage of viral vectors. Lentiviral third-generation self-inactivating LeGO vectors [143] designed for identification and quantification of live cell clones in vitro and in vivo using flow cytometry were used for the optical barcoding of two cell lines derived from malignant glioma, from which clones were injected in vivo in the mouse striatum to study tumor heterogeneity. The authors showed that distinct clones have variable doubling times in vivo [144].

### Injury

The hGFAP-StarTrack strategy was used to illustrate the astrogliosis clone response to acute multifocal needle injury in the adult mouse cerebral cortex in vivo [145]. One week after trauma, heterogeneity of reactivity, predominantly between clones and occasionally among the same clone was observed, suggesting a differential response of astrocytes due to distinct cellular sources [145]. Unicolor pairs of reactive astrocytes were also observed in R26R-Confetti; GLAST-Cre^ERT2^ mouse cortex following a single stab wound injury, suggesting that specialized astrocyte subtypes might proliferate in response to brain damage [30]. A clone was then defined as a group of proliferating cells sharing the same color with spacing between sibling cells of less than 5 µm [31]. Interestingly, larger astrocyte clones were described after a second stab wound injury, suggesting that initially quiescent clones of astrocytes can proliferate in response to a second trauma but not after the first one [31]. However, clonal analysis of Confetti-labeled cells in the intact or injured nervous system is only achievable with a sparse labeling and after amplification of the weak endogenous signal. As 3 out of 4 native FP are antigenically indistinguishable after immunostaining, two colors are finally exploitable on fixed tissue. Thus, the use of the R26R-Confetti reporter mouse to study clonal relationships in the nervous system should be limited to slowly proliferating cells and poorly migrating sibling cells in three-dimensional tissue. Also of note is that the MADM genetic labeling strategy has been applied to sparsely label reactive astrocytes after spinal cord injury in MADM; GFAP-Cre mice and has shown that interacting and probably overlapping reactive astrocytes are only observed at the scar border [146]. The hGFAP-StarTrack approach, combined with immunohistochemistry against K_v_4.3a voltage-dependent potassium channel, has revealed functional subpopulations of astrocytes depending on their ontogenetic origin [147].

### Multiple sclerosis

The same hGFAP-StarTrack strategy was applied to determine the reaction of clonally related astrocytes to cortical damage caused by experimental autoimmune encephalomyelitis (EAE) [148]. As described after mechanical brain injury [145], various clonal responses were observed in or close to perivascular inflammatory infiltrates around the peak of the disease [148]. Sibling astrocytes are most likely to respond homogeneously to inflammatory demyelinating lesions by adopting the hypertrophic hallmarks of reactive astrocytes, or to some extent keeping an unresponsive protoplasmic morphology characteristic of resting astrocytes. However, mixed clones formed by reactive and non-reactive sibling cells were also described, suggesting that astrocyte response diversity is both developmentally established and regulated by the molecular environment [148]. In addition, the response of NG2-glial clones derived from embryonic GFAP-positive progenitor cells to EAE lesions has been addressed by recycling these previous animals and tissues [149]. In inflammatory and lesioned mouse cortex, NG2-glial clones were spatially distributed in different areas and most of them were located in the lower cortical layers [149]. Similar morphological diversities were observed for NG2-glia and GFAP clones after both mechanical and demyelinating damages. Robin Franklin’s laboratory used Confetti; Sox10-iCre mice to investigate the individual response of peri-lesional OPC to focal toxin-induced demyelination. After identification of clones with hierarchical clustering analysis, the authors determined that the number and size of clones progressively increase from 3–21 days after the lesion, suggesting that many surrounding cells are responding to the lesion by repopulating it and undergoing few cycles of division [150].

## Imaging solutions and new software tools for quantitative analysis of multicolor data sets

As described previously, multicolor strategies are useful for investigating numerous biological processes in various tissues. Now further progress using multicolor models relies largely on overcoming challenges in the field of imaging. Tracking the morphology and movements of individual cells in densely packed tissue is achievable for only a fraction of these cells located on the surface or at the periphery of the tissue. It can be done using mostly high magnification imaging performed on fixed or thin live sections, followed by manual or semi-automated segmentation. However, it severely restricts the quantity of analyzable cells per sample. Similarly, a fair assessment of lineage and connectivity of single-color cells can be done only by restricting the number of analyzed cells that should be sparsely arranged in a tissue. To resolve these issues, advanced multicolor imaging techniques of large and possibly live samples are required. Here we provide an overview of equipment and techniques that improve multicolor data collection and analysis.

### Two- and three-photon-excited multicolor fluorescence microscopy (2P, 3P)

Three-dimensional imaging over several hundreds of micrometers can be achieved by two-photon (2P) microscopy. For example, multicolor 2P imaging combined with cell type specific (red and green) and senile plaque (cyan) fluorescent markers was used to study a mouse model of Alzheimer disease in vivo [151]. Moreover, brains electroporated in utero with Brainbow transgenes expressing mCerulean, mEYFP and tdTomato (and/or mCherry), live Brainbow-labeled chicken embryonic tissue and live developing multicolor Drosophila embryos were successfully imaged with a high resolution at adult stages using 2P wavelength mixing obtained by spatial and temporal overlapping of two pulse trains produced by a femtosecond laser and an optical parametric oscillator [45]. This method was further implemented by combining wavelength mixing and (1) 2P scanned light-sheet illumination, which was successfully applied to the time-lapse imaging of the beating heart of multicolor zebrafish embryos [152], and (2) serial block-face image acquisition [47]. ChroMS microscopy provides 3D multicolor and label-free nonlinear signals imaging of transgenic, electroporated or virus-injected mouse brains over several mm^3^ continuous volumes or brain-wide at discrete axial positions, with micron-scale resolution and submicron channel coregistration. This strategy enabled the color-based 3D analysis of astrocyte morphology and contacts in the mouse cerebral cortex and the tracing of individual pyramidal neurons in densely MAGIC Markers-labeled tissue, as well as multiplexed whole-brain mapping of axonal projections labeled with spectrally distinct viral tracers. ChroMS on MAGIC Markers-labeled cortices was subsequently performed to analyze the dispersion of clonally related astrocytes arising from distinct neighboring mouse cortical progenitors, their 3D cellular morphology and spatial arrangement [46]. However, 2P imaging at large depths becomes limited by increased out-of-focus background from the excitation cone as one increases the power at the tissue surface to compensate for tissue scattering. The use of three-photon (3P) microscopy allows deeper imaging with a wavelength window (1300–1700 nm) higher than the 2P one (700–1100 nm), providing a better combination of tissue scattering and absorption properties. Simultaneous 3P excitation of GFP, mRFP/mCherry/tdTomato and label-free nonlinear signals was applied to fixed mouse brain, live developing chick spinal cord, and live adult zebrafish brain imaging, with superior contrast at large depths compared to 2P [153], opening the way to higher dimensions of multicolor large volume imaging.

### Tissue clearing of multicolor brain samples

As 2P, 3P and confocal microscopy are limited in tissue penetration, and stitching of serial block-face volumes can be time consuming and technically challenging, multiple tissue clearing strategies have been developed (reviewed in [154]) and successfully applied to various tissues. Each method has distinct clearing effects, requires different incubation times, can lead to various tissue size deformation, and results in variable fluorescence preservation and imaging depth [155]. Moreover, they are often not compatible with the preservation of several sensible endogenous FP over imaging time due to the use of detergents, solvents or heating. Immunostaining of these FP can lead to insufficient antibody tissue penetration or lack of antibody specificity for FP with high sequence homology. Nevertheless, several strategies worked on multicolor samples with endogenous fluorescence. The Scale approach [156] has been used to clear adult Zebrabow telencephali, and to perform Brainbow clonal analysis on whole-mount samples to show, in 3D, the contribution of individual embryonic radial glial cells to the adult pallium [157]. In mice, the best current clearing protocols preserving endogenous fluorescence obtained with classic multicolor strategies are the SeeDB2 [158] and CUBIC protocols [159–161]. The SeeDB2 approach is based on iohexol/saponin (See Deep Brain 2, improved from SeeDB, itself based on fructose/thioglycerol [162, 163]). CUBIC protocols, on the other hand, derive from a variant of Scale that includes a first reagent based on aminoalcohol/urea solution and a second one based on a sucrose/triethanolamine/urea solution.

Also in mouse, Sakaguchi and colleagues developed an improved multicolor labeling method named Tetbow [57] which enhances FP expression levels with a tetracycline-operator system. They optimized Tetbow for either plasmid or AAVs vector-mediated multicolor labeling, applied it in the mouse brain by in utero electroporation or stereotaxic injections and combined it with SeeDB2 tissue clearing [158]. It enabled them to visualize the 3D architecture of individual adult multicolor neurons, including dendritic spines and the axonal projections of individual mitral/tufted cells along several millimeters from the olfactory bulb to the olfactory cortex. As expression of Tetbow AAVs can lead to toxic effects on cellular functions after a prolonged incubation period, as well as lipid-rich myelinated axons are challenging to clear without quenching endogenous FP, Sakaguchi and colleagues modified their system with genetically encoded chemical tags labeled with synthetic fluorophores. These chemical tags remained stable after SeeDB2, BABB and 3Disco clearing, offering a good alternative for future large volume multicolor studies [57]. Outside of the brain, the clearing agent FUnGI (Fructose, Urea, and Glycerol for Imaging) allowed high-resolution large-scale confocal and multiphoton imaging of intact solid Confetti-labeled tumors in mouse mammary glands [164, 165]. In addition, the same group used intravital microscopy which allows cell behavior and dynamic observation in live tissue, to study single-cell dynamics in mammary gland structures using dissected Confetti samples in imaging chambers [166]. Using this method, they performed single cell and tissue microenvironment time-lapse imaging and lineage-tracing in multicolor Confetti samples with additional Far-red dyes or endogenous signals such as collagen second harmonic generation (SHG). Using specific filters and sequential excitation with dual multiphoton lasers before fixation, immunostaining and FUnGI procedure to correlate multiphoton with high-resolution confocal imaging, images could then be visualized in 3D using Imaris software with 3D Cyan/Red glasses [166].

### Expansion microscopy

Besides hydrophilic-based tissue clearing methods such as CUBIC and SeeDB2, some hydrogel-based tissue clearing approaches are also suitable to image endogenous or amplified FP. Derivatives of expansion microscopy (ExM) and in particular protein retention (proExM), iterative (iExM) and multi-round immunostaining ExM (miriEx) have been successfully used together with multicolor labeling. These powerful histological methods increase the effective resolution to nanoscale through both isotropic expansion (physical swelling from ~ 4 to 5-fold with proExM and miriEx to ~ 16–22-fold after iExM treatment) and optical clearing of the tissue. Edward Boyden’s laboratory optimized its original ExM protocol composed of 3 major steps (i.e. gelation, proteinase K digestion and expansion in water) to overcome the initial endogenous fluorescence loss by anchoring genetically encoded FP directly to the swellable gel through active ester pretreatment before the classical ExM pipeline [126]. This proExM protocol preserves more than 50% of the native fluorescence intensity of the majority of twenty commonly used FP addressed to the nucleus of cultured HEK-293 cells, such as EBFP2, mTurquoise2, ECFP, mTFP1, EGFP, EYFP, tdTomato, and mCherry present in several constructs described above [126]. To further resolve any nanoscale organization with even epifluorescence microscope (bringing the resolution from ~ 70 nm with proExM protocol to ~ 25 nm), the iExM methodology adds a second round of expansion by re-embedding the hydrogel-tissue hybrid, followed by re-amplification of the signal [129]. Recently developed, miriEx combines one expansion step followed by multiplexed staining on Brainbow labeled tissue to simultaneously profile the morphology, molecular subtype identity and putative synaptic connectivity between intermingled neurons in a single mammalian brain section [56]. At each staining round, the expanded tissue undergoes a labeling, imaging using the EYFP channel as fiducial marker and stain removal step [56]. Unprecedented details of connectomics have also been collected from expanded mouse brain labeled with Brainbow AAVs [56, 126, 129] and transgenic Bitbow drosophila [127]. The enhancement of the multicolor signal by immunostaining is, however, required to compensate the signal reduction induced by physical expansion [56, 126, 127, 129].

### FACS

An alternative way to circumvent time-consuming imaging and quickly and quantitatively assess clonality is the fluorescence-activated cell sorting (FACS) method. Several attempts had been made to sort out lymphocytes, hematopoietic stem cells or skin squamous cancer cells by their color clonotype using the multicolor Confetti reporter mouse. Confetti cells express a reduced palette of colors in comparison to other multicolor reporters and the distinct subcellular localization of spectrally adjacent GFP and YFP can be hard to discriminate with the classical flow cytometer set up. Independently of sequencing, Martinez and colleagues simultaneously tracked up to 8 distinct T cell clonotypes during an autoimmune response in Rosa26Cre^ERT^; Confetti mice after sparse labeling using a traditional flow cytometer [167]. Of note, due to the suboptimal wavelength of excitation at 405 and 488 nm, CFP clones might actually have been interpreted as GFP or YFP clones and therefore CFP clones were not directly detected as stated by the authors [167]. In a later study, Reeves and colleagues have investigated clonal dynamics progression of carcinoma by combining multicolored lineage tracing after local application of tamoxifen on the Confetti; K5-Cre^ERT^ back skin followed by carcinogen treatment, FACS separation of colored clones and Sanger sequencing [25]. In this case, both GFP + and YFP + clones were gated together during the separation process with a regular FACS due to the spectral overlap of GFP and YFP. Nevertheless, they demonstrated that skin benign tumors have a polyclonal composition with only one colored clone deriving from a single initiated mother cell carrying the oncogenic mutation. Stable benign papillomas retain this multiclonal composition while malignant carcinomas are characterized by the existence of a dominant clone [25]. Hagert and colleagues recently developed a high-throughput method using both flow and imaging flow cytometry to evaluate Confetti color distribution in lymphocytes [23]. Isolated from tamoxifen induced Confetti; CD4-Cre^ERT2^ lymphoid organs, live singlet cells within the appropriate size and granularity range were classically selected on CD4 positivity. The cells were then successively segregated based on their different fluorescence ratio and their subcellular localization was determined as well using an imaging flow cytometer. In hemizygous animals, the 4 FP are rapidly and easily recognizable using both flow cytometer types. Moreover, 9 out of 10 color combinations were unambiguously discriminated in homozygous mice, even after manual visual inspection of the colored cells during imaging flow cytometry. Despite the generation of subcellular masks and appropriate fluorochrome compensation, it is still challenging to distinguish between GFP + YFP + and YFP + YFP + populations with a single common wavelength of excitation.

### Automated analysis

As manual analyses of multicolor images can lead to errors due to subjective bias, Ortiz-Alvarez and colleagues developed an automatic color analysis method of multicolor ependymal cells after ventricular (V)-subventricular (SVZ) electroporation of Nucbow MM at E14.5 and Foxj1 immunostaining at P15-P20 [106]. Cells were segmented using the smooth manifold extraction tool (SME, [168]) on the FoxJ1 + signal before automatic extraction of color content information (saturation, value, hue in the RGB tridimensional space) and 3D spatial distances. This method enabled the authors to study the lineage of ependymal cells and to show that most ependymal cells derive from 3 or less cell divisions following the labeling of radial glial cells at E14.5 and that they originate from either one terminal symmetric or asymmetric division. Based on StarTrack labeled images, Salvi and collaborators have developed a different method: the Fluorescent cell Analysis Segmentation Tool (FAST) which is an adaptive, fully automated and multichannel method for the automatic segmentation of neural cells in fluorescent microscopy images and for their classification into clones [169]. Developed by Ghigo and collaborators, the analysis software “ClusterQuant” enables to determine the clustering index of CFP + cells in Ubow multicolor mice, is specifically designed for statistical analysis of colored cell spatial distribution, and can be applied to any multicolor fate mapping system [17]. Using ClusterQuant analysis software, Ubow cell types are manually labeled within microscopic images. The subsequent automatic computation of the corresponding Voronoi diagrams is then combined with Monte Carlo simulations to obtain a quantitative statistical measure of the spatial distribution (i.e., cluster formation or diffuse cell spreading) of FP-expressing cells [17]. Previously used for analysis of cell clusters in single Ubow + tissue planes, a 3D version of the ClusterQuant software has been developed and used to explore the tissue clonality of the conventional dendritic cells (cDC). These cDC are a type of leukocytes that play a key role in innate immunity and form a network of immune sentinel cells in most tissues of mice and humans. Cabeza-Cabrerizo and collaborators have developed a multicolor Rosa26 Confetti-based genetic model coupled to flow cytometric spectral analysis which allows separation of closely related fluorophores, including GFP and YFP to label individual cDC progenitors and pre-cDC with one of four possible fluorophores. Using this strategy associated to image analysis and 3D cluster quantification with ClusterQuant, Cabeza-Cabrerizo and collaborators have studied how cDC precursors seed tissues at the single-cell level in the absence of cell transfer. They also showed that pre-cDC in the steady state enter peripheral tissues and can divide locally before differentiating into cDC, which display residual proliferative capacity [170].

## Conclusion

Multicolor strategies applied to stem cell lineage, cellular plasticity and pathogenesis studies offer a wide range of essential tools to address fundamental issues of tissue development, renewal and physiopathology on a multicellular scale. These approaches make possible to characterize the properties of embryonic and adult stem cells as well as their individual contributions to the morphogenesis and homeostasis of numerous tissue models.

However, the usefulness of these multicolor approaches relies on a clearly defined assessment of the scope of their use beforehand. Indeed, depending on basic biological processes investigated (clonal output vs tissue homeostasis), the complexity of the tissue of interest (2D vs 3D expansion) and the quantity of cells that need to be individualized and tracked concomitantly, the experimenter will have to select carefully the most appropriate multicolor model. Several technical considerations will also play a part in this choice, such as the need for real-time monitoring of biological phenomena that will require a strong expression of endogenous fluorescence signal without resorting to signal amplification using immunolabeling. In addition to the accessibility of the progenitor targeted population in the tissue of interest, the required spatiotemporal control of the marked population will lead the experimenter to turn either to strategies based solely on intercrossing multicolored transgenic mice with mice expressing an inducible form of recombinase Cre downstream of a specific promoter, or to acute transgenesis approaches based on physical targeting of progenitors by electroporation at a given stage of development (Table 4).

In conclusion, these multicolor models offer tremendous opportunities to investigate complex biological questions involving tracking and analysis of multiple neighboring cells simultaneously. These powerful, yet complex tools that are rapidly becoming increasingly versatile will reach a growing audience, as more high-throughput multichannel imaging solutions and efficient automated 3D color tracking software tools become available.

## Data Availability

Not applicable.
